# Repetitive transcranial magnetic stimulation regulates effective connectivity patterns of brain networks in the spectrum of preclinical Alzheimer’s disease

**DOI:** 10.3389/fnagi.2024.1343926

**Published:** 2024-02-12

**Authors:** Xuhong Liang, Chen Xue, Darui Zheng, Qianqian Yuan, Wenzhang Qi, Yiming Ruan, Shanshan Chen, Yu Song, Huimin Wu, Xiang Lu, Chaoyong Xiao, Jiu Chen

**Affiliations:** ^1^Department of Radiology, The Affiliated Brain Hospital of Nanjing Medical University, Nanjing, China; ^2^Department of Neurology, The Affiliated Brain Hospital of Nanjing Medical University, Nanjing, China; ^3^Department of Neurology, Northern Jiangsu People's Hospital, Clinical Medical College, Yangzhou University, Yangzhou, China; ^4^Department of Radiology, Nanjing Drum Tower Hospital, Affiliated Hospital of Medical School, Nanjing University, Nanjing, China; ^5^Institute of Medical Imaging and Artificial Intelligence, Nanjing University, Nanjing, China; ^6^Medical Imaging Center, Nanjing Drum Tower Hospital, The Affiliated Hospital of Nanjing University Medical School, Nanjing, China

**Keywords:** subjective cognitive decline, amnestic mild cognitive impairment, repetitive transcranial magnetic stimulation, dynamic causal model, effective connectivity

## Abstract

**Objectives:**

Subjective cognitive decline (SCD) and amnestic mild cognitive impairment (aMCI) are considered as the spectrum of preclinical Alzheimer’s disease (AD), with abnormal brain network connectivity as the main neuroimaging feature. Repetitive transcranial magnetic stimulation (rTMS) has been proven to be an effective non-invasive technique for addressing neuropsychiatric disorders. This study aims to explore the potential of targeted rTMS to regulate effective connectivity within the default mode network (DMN) and the executive control network (CEN), thereby improving cognitive function.

**Methods:**

This study included 86 healthy controls (HCs), 72 SCDs, and 86 aMCIs. Among them, 10 SCDs and 11 aMCIs received a 2-week rTMS course of 5-day, once-daily. Cross-sectional analysis with the spectral dynamic causal model (spDCM) was used to analyze the DMN and CEN effective connectivity patterns of the three groups. Afterwards, longitudinal analysis was conducted on the changes in effective connectivity patterns and cognitive function before and after rTMS for SCD and aMCI, and the correlation between them was analyzed.

**Results:**

Cross-sectional analysis showed different effective connectivity patterns in the DMN and CEN among the three groups. Longitudinal analysis showed that the effective connectivity pattern of the SCD had changed, accompanied by improvements in episodic memory. Correlation analysis indicated a negative relationship between effective connectivity from the left angular gyrus (ANG) to the anterior cingulate gyrus and the ANG.R to the right middle frontal gyrus, with visuospatial and executive function, respectively. In patients with aMCI, episodic memory and executive function improved, while the effective connectivity pattern remained unchanged.

**Conclusion:**

This study demonstrates that PCUN-targeted rTMS in SCD regulates the abnormal effective connectivity patterns in DMN and CEN, thereby improving cognition function. Conversely, in aMCI, the mechanism of improvement may differ. Our findings further suggest that rTMS is more effective in preventing or delaying disease progression in the earlier stages of the AD spectrum.

**Clinical Trial Registration:**

http://www.chictr.org.cn, ChiCTR2000034533.

## 1 Background

Amnestic mild cognitive impairment (aMCI) is a pre-dementia syndrome marked by declining objective cognitive function declines, while daily life functionality remains intact (Winblad et al., 2004). Subjective Cognitive Decline (SCD) refers to an individual’s subjective perception of cognitive decline in the absence of objective impairment on cognitive assessments, and is considered a potential prodromal manifestation of MCI (Rabin et al., 2017). A growing body of research indicates that SCD and aMCI continue to impair brain function and structure and accelerate the progression of Alzheimer’s disease (AD) to varying degrees (Mishra et al., 2018; Xue et al., 2019). These observations underscore the notion SCD and aMCI can be considered precursors to AD, sharing underlying therapeutic mechanisms while existing in different pathological states at different stages of the disease (Xue et al., 2019). Given the profound impact of severe cognitive deterioration on an individual’s daily life, it is essential to prevent or delay pathological cognitive decline and determine effective treatment options for the spectrum of preclinical AD.

To date, pharmacological interventions have demonstrated limited efficacy in treating AD, necessitating the exploration of other treatments (Livingston et al., 2017). Repetitive transcranial magnetic stimulation (rTMS) is the most widely researched non-invasive brain stimulation technique that has been applied to a variety of psychiatric disorders (Sabbagh et al., 2020). The research indicates that low-frequency rTMS can reduce excitability in the target cortical area, while high-frequency rTMS can enhance excitability in the target cortical area (Maeda et al., 2000). rTMS involves the application of coils to the scalp to modulate underlying brain activity by generating a magnetic field that surrounds cortical neurons, using a strong but brief electromagnetic pulse (George and Aston-Jones, 2010). Previous studies on neuromodulation have confirmed the ability of rTMS acting on the dorsolateral prefrontal cortex (DLPFC) to enhance overall cognitive function and improve clinical performance (Cotelli et al., 2011; Bagattini et al., 2020). Several recent studies have underscored the potential of rTMS targeted at the precuneus (PCUN) to enhance memory and attenuate cognitive decline in preclinical AD (Chen et al., 2017, 2021). The PCUN is a key brain region in AD that undergoes pathological changes 10–20 years before the manifestation of clinical symptoms (Bateman et al., 2012). These alterations include decreased cerebral blood flow (CBF), amyloid deposits, cortical thickness, and functional connectivity (FC) (Ikonomovic et al., 2011; Chen et al., 2017; Thomas et al., 2019). Therefore, it is reasonable to believe that the PCUN is a vulnerable region within the spectrum of preclinical AD, rendering it an ideal target for therapeutic intervention.

Current studies on the spectrum of preclinical AD using functional magnetic resonance imaging (fMRI) mainly focus on the dynamics of brain networks. The default mode network (DMN) and the executive control network (CEN) are the two core brain networks (Chen et al., 2013). The DMN is mainly located in the ventromedial prefrontal cortex and the posterior cingulate cortex. It plays an important role in the self-reference psychological process and social functioning, exhibiting increased activity in internally oriented behaviors (Broyd et al., 2009). In contrast, the CEN is mainly located in the DLPFC and plays an important role in the active maintenance and operation of working memory information, showing increased activity in externally directed behaviors (Bressler and Menon, 2010). The FC is an important tool for studying brain networks, measuring internal fluctuations in blood oxygenation level-dependent signals in different brain regions and analyzing the cooperation between different brain regions during rest or task execution (Zhu et al., 2021). Numerous studies have shown changes in the FC of the DMN and CEN, accompanied by changes in cognitive function (Xu et al., 2020; Yuan et al., 2021).

Recent research on the impact of rTMS on brain networks has mostly focused on the changes in FC within these networks (Cotelli et al., 2011; Pilato et al., 2012). However, FC is based on the correlation between the time series of brain regions, thus lacking the ability to provide the directionality of inter-regional brain interactions; hence, it does not represent real “connectivity” (Friston et al., 2003). Therefore, FC cannot accurately map specific changes in brain networks or provide an in-depth understanding of changes in brain activity.

Effective connectivity is mostly model-based, and it partially addresses the limitations of traditional data analysis by elucidating the causal effects among brain regions and emphasizing the direction of action of these interactions. Among all the effective connectivity methods, the dynamic causal model (DCM) shows superior performance in neuronal coupling modeling (Friston, 2009). Initially designed for task-state fMRI, DCM was subsequently adapted by Friston et al. (2003) to estimate effective connectivity from resting-state fMRI time series under the smoothness assumption of cross spectra, resulting in the formulation of the spectral dynamic causal model (spDCM). SpDCM constructs a reliable model of coupling neuron states and generates complex cross spectra to measure directional neural effects (Friston et al., 2014). Relevant studies have shown changes in the effective connectivity of brain networks in the spectrum of preclinical AD, and these changes have shown correlations with cognitive function, revealing the potential neural processes in the course of the disease (Chand et al., 2017, 2018). However, it remains unclear whether rTMS interventions can regulate the effective connectivity of brain networks and thus improve cognition.

Therefore, we aimed to validate the hypothesis that PCUN-targeted rTMS could improve cognitive function in the spectrum of preclinical AD by regulating the effective connectivity patterns within brain networks. First, we performed a cross-sectional analysis using spDCM to evaluate the causal interactions within the DMN and CEN and compared the effective connectivity patterns observed in patients with SCD, aMCI, and healthy controls (HCs) to investigate potential pathological changes in the effective connectivity patterns of brain networks in the spectrum of preclinical AD. In addition, we introduced a PCUN-targeted rTMS intervention, assuming that this treatment might change the effective connectivity patterns of the DMN and CEN networks, thereby improving cognitive function.

## 2 Methods and materials

### 2.1 Subjects

The data utilized in this study were obtained from our internal database, Nanjing Brain Hospital-Alzheimer’s Disease Spectrum Neuroimaging Project 2 (NBH-ADsnp2) (Nanjing, China), which is continuously updated. For information on NBH-ADsnp2, see Supplementary material. A total of 256 subjects (89 with HC, 75 with SCD, and 92 with aMCI) were initially enrolled in this study. However, 12 participants (3 with HC, 3 with SCD, and 6 with aMCI) with excessive head movements (cumulative translation or rotation >3.0 mm or 3.0°) were excluded from the analysis. Thus, a total of 244 subjects (86 with HC, 72 with SCD, and 86 with aMCI) were finally included in the study. Of these, 10 patients diagnosed with SCD and 11 patients diagnosed with aMCI underwent follow-up rTMS intervention, fMRI, and clinical cognitive data acquisition. The specific inclusion and exclusion criteria for the subjects are described in Supplementary material S1.

### 2.2 Ethics approval statement

This study was approved by the Human Participants Ethics Committee of the Affiliated Brain Hospital of Nanjing Medical University (No. 2018-KY010-01 and No. 2020-KY010-02). Written informed consent was obtained from all subjects prior to enrolment.

### 2.3 Neuropsychological assessments

A standardized clinical interview and comprehensive neuropsychological assessment were conducted in this study. The comprehensive neuropsychological assessment was divided into four cognitive domains: episodic memory (AVLT, LMT, and CFT-20-min-DR), information processing speed (DSST, TMT-A, Stoop-A, and Stoop-B), visuospatial function (CFT and CDT), and executive function (TMT-B, Stoop-C, DST-backward, VFT, and Semantic Similarity). Detailed information for each neuropsychological assessment can be found in Supplementary material S2.

### 2.4 MRI data acquisition

All magnetic resonance imaging (MRI) data were acquired at the Affiliated Brain Hospital of Nanjing Medical University using a 3.0 Tesla Verio Siemens scanner equipped with an 8-channel head coil. Resting-state functional images were acquired when participants were instructed to rest with their eyes open, to not fall asleep, and to not think of anything in particular. The gradient-echo echo-planar imaging (GRE-EPI) sequence included 240 volumes. The parameters were: repetition time (TR) = 2,000 ms, echo time (TE) = 30 ms, number of slices = 36, thickness = 4.0 mm, gap = 0 mm, matrix = 64× 64, flip angle (FA) = 90°, field of view (FOV) = 220 mm × 220 mm, acquisition bandwidth = 100 kHz, and voxel size = 3.4 × 3.4 × 4 mm^3^.

High-resolution T1-weighted images were acquired by 3D magnetization-prepared rapid gradient-echo (MPRAGE) sequence. The parameters were: TR = 1,900 ms, TE = 2.48 ms, inversion time (TI) = 900 ms, number of slices = 176, thickness = 1.0 mm, gap = 0.5 mm, matrix = 256 × 256, FA = 9°, FOV = 256 mm × 256 mm, and voxel size = 1 × 1 × 1 mm^3^.

### 2.5 fMRI data preprocessing

All fMRI data were preprocessed using MATLAB 2013b and DPABI software (Yan et al., 2016). As a preliminary step, the first 10 volumes were discarded to reduce the instability of the MRI signal. Subsequently, slice timing correction and head motion correction were performed (Power et al., 2012; Van Dijk et al., 2012). Interscan motion was assessed with translation/rotation, and an exclusion criterion (>3 mm translation and/or > 3^°^ rotation in each direction) was set. The functional image of each subject was aligned with their respective structural image. Next, to spatially normalize the fMRI data, the T1 images were segmented into gray matter (GM), white matter (WM), and cerebrospinal fluid (CSF) using the Diffeomorphic Anatomical Registration Through Exponentiated Lie Algebra (DARTEL) algorithm. Then, the functional images were normalized by DARTEL to the MNI space (resampling voxel size, 3 × 3 × 3 mm^3^). Finally, spatial smoothing was applied using a Gaussian kernel with a full-width at half maximum of 6 mm^3^ to reduce spatial noise. Independent component analysis (ICA) was performed on the pre-processed data. To maintain the independence of the data during the ICA and capture the intrinsic independent components for more accurate analysis results, we refrained from performing covariate removal during the data preprocessing stage.

### 2.6 Group independent component analysis and regions of interest

The number of independent components in ICA analysis is typically not fixed and depends on the research question and data characteristics. In this study, we employed the informix algorithm available in the Group ICA toolbox^1^ to decompose the data into 27 independent components. Subsequently, these components were subjected to spatial correlation analysis against the corresponding network templates provided by Smith et al. (2009) and Xue et al. (2021). According to the results of the correlation calculation, the independent components were sorted, and the components that showed the highest alignment with the DMN and CEN templates were selected for further analysis.

After selecting the independent components of the DMN and CEN, we used the XjView toolbox^2^ to obtain the peak coordinates of the core regions of the DMN and CEN. Based on the obtained core peak coordinates, a total of 13 ROIs were defined as spheres with a radius of 6 mm in this study. The eight ROIs of the DMN were: the PCUN, bilateral ANG, ACG, left MFG, left middle temporal gyrus (MTG), and bilateral inferior occipital gyrus (IOG). The five ROIs of the CEN were: the right ANG, left inferior parietal lobule (IPL), left MTG, and bilateral MFG.

### 2.7 Spectral dynamic causal modeling

Using SPM,^3^ we established a general linear model (GLM) for each subject and regressed the extracted CSF, WM signals, and motion parameters as covariates to adjust the GLM. Due to its extensive validation and widespread use, the Jenkinson motion parameters have been considered a standardized and accurate method for precise measurement of head motion. Therefore, we chose to include the Jenkinson motion parameters as covariates in our analysis. Subsequently, a sphere model was established for the selected ROIs, and a GM mask was used to assist in extracting the time series from each ROI. Next, an 8 × 8 fully connected model for DMN networks and a 5 × 5 fully connected model for CEN networks were established respectively, and cross spectra were used for parameter estimation. We were modeling on the resting-state fMRI data. No exogenous input was included in the model. After parameter estimation was completed, all possible models were defined. Bayesian estimation was applied to obtain the corresponding posterior probabilities for each model. The optimal model was finally obtained, along with its effective connectivity patterns.

### 2.8 rTMS protocol

This study used a Magstim Rapid2 magnetic stimulator connected to a 70-mm figure-8-shaped coil with two coils cross-tipped at the Pz site of the electroencephalogram system 10–20, targeting the PCUN region. The stimulus intensity was set to 100% of the resting-state motor threshold (MT). MT was determined by applying rTMS to the left PCUN (located approximately above the central sulcus) and shifting it by 0.5 cm along the scalp to the left motor cortical area (M1) (Koch et al., 2018). At the same time, the opposite side (right) was monitored to ensure relaxation of the first dorsal interosseous muscle. At least 5 of the 10 experiments produced a minimum intensity value of 50 μV motor evoked potential (MEP). We identified the interpupillary point as the anatomical landmark on the scalp and matched it with the corresponding marker on the headgear, establishing a correspondence between them. Subsequently, based on the calibrated headgear device, we determined the target region of the PCUN.

A total of 10 patients with SCD and 11 patients with aMCI underwent a total of 25 sessions of rTMS. Continuous stimulation for 40 times, each lasting 4 s, followed by an interval of 56 s. This sequence was repeated for 25 rounds, resulting in a total of 1,000 pulses, and lasting for 25 min. Each subject received this treatment once a day, five days a week (Monday to Friday), for a duration of two weeks per course of treatment. The entire procedure was repeated for a total of four weeks, equivalent to two treatment courses (Chen et al., 2021; Yuan et al., 2023). Subsequently, imaging data acquisition and neuropsychological evaluation were performed for each subject.

### 2.9 Statistical analyses

The Statistical Package for the Social Sciences (SPSS) software version 22.0 (IBM, Armonk, NY, United States) was used for statistical analyses. The analysis of variance (ANOVA), paired *t*-test, and the chi-square test were conducted to compare the demographic and neurocognitive data among groups, namely the HCs, and patients with SCD, aMCI, and SCD and aMCI before rTMS and after rTMS. Bonferroni correction was used for *post hoc* comparisons. A *p*-value < 0.05 indicated statistically significant differences.

For the 8 × 8 fully connected model built in the DMN network and the 5 × 5 fully connected model built in the CEN network, the effective connectivity values for all subjects were obtained. These values were subjected to statistical testing using one-sample *t*-test (*p* < 0.05) to find effective connectivity links that significantly deviated from zero within the DMN and CEN networks for each of the three groups of subjects. Next, ANOVA was used to compare the effective connectivity patterns among the HC, SCD, and aMCI groups (*p* < 0.05, uncorrected) to identify any differences in the effective connectivity between the groups. Then, after controlling for the effects of age, gender, and level of education, correlation analyses were conducted between changes in effective connectivity and cognitive function to reveal their relationships (*p* < 0.05).

After conducting rTMS intervention in 10 patients with SCD and 11 patients with aMCI, paired *t*-tests were used to compare the changes in effective connectivity before and after rTMS treatment in the SCD and aMCI groups (*p* < 0.05, uncorrected). Similarly, correlation analyses were conducted to explore associations between changes in effective connectivity and cognitive changes before and after rTMS intervention (*p* < 0.05).

## 3 Results

### 3.1 Demographic and neurocognitive characteristics

The demographic and neurocognitive information of all subjects is detailed in Tables 1, 2. Detailed raw scores of individual neuropsychological tests for all subjects and the *p*-values of demographics and clinical measures across different groups in Supplementary Tables S1–S3. We found no significant difference in age and gender among the three groups of patients, whereas the educational level of patients with aMCI was significantly lower compared to HCs. In addition, the aMCI group showed a decline in episodic memory, information processing speed, executive function, and visuospatial function, compared to the HC and SCD groups. After receiving rTMS intervention, the SCD group showed improved episodic memory, and the aMCI group showed improved episodic memory and executive function.

**Table 1:** Demographics and clinical measures of HC and patients with SCD, and aMCI.

Characteristics	HC	SCD	aMCI	*F*-values(χ^2^)	*p*-values
	*n* = 86	*n* = 72	*n* = 86		
Age (years)	63.35(7.010)	65.28(7.527)	65.24(7.521)	1.861	0.158
Gender (male/female)	32/54	17/55	29/57	3.520	0.172
Education level (years)	12.35(2.683)	11.73(2.696)	11.04(2.845)^b^	4.886	0.008^**^
Composite *Z* scores of each cognitive domain					
Episodic memory	0.24(0.61)	0.28(0.59)	−0.48(0.63)^bc^	41.266	0.000^***^
Information processing speed	0.23(0.74)	0.17(0.70)	−0.37(0.68)^bc^	18.568	0.000^***^
Executive function	0.25(0.48)	0.19(0.47)	−0.41(0.50)^bc^	49.080	0.000^***^
Visuospatial function	0.15(0.72)	0.13(0.68)	−0.26(0.88)^bc^	7.797	0.001^***^

Data are presented as mean (standard deviation, SD). HC, healthy controls; SCD, subjective cognitive decline; aMCI, amnestic mild cognitive impairment. *Significant differences were found among HC, SCD, and aMCI subjects. *p ≤ 0.05, ** *p* ≤ 0.01, ***p ≤ 0.001. Most *p*-values were obtained using ANOVA, except for gender (chi-square test). Comparisons of each paired group were conducted to further reveal the source of ANOVA difference (b: aMCI vs. HC; c: aMCI vs. SCD). Bonferroni correction was applied for multiple group comparisons. This study utilized the composite *Z* scores to determine the level of each cognitive domain.

**Table 2:** Demographics and clinical measures of SCD and aMCI before rTMS and after rTMS.

Characteristics	Before rTMS SCD	After rTMS SCD	*T*-values (χ^2^)	*p*-values	Before rTMS aMCI	After rTMS aMCI	*T*-values (χ^2^)	*p*-values
	*n* = 10	*n* = 10			*n* = 11	*n* = 11		
Age (years)	66.00(7.874)	67.00(7.513)	−2.2535	0.032*	65.82(7.534)	66.45(7.647)	−1.750	0.111
Gender (male/female)	3/7	3/7	0.000	1.000	2/9	2/9	0.000	1.000
Education level (years)	11.40(3.273)	11.40(3.273)	0.000	1.000	12.55(3.197)	12.55(3.197)	0.000	1.000
Composite *Z* scores of each cognitive domain								
Episodic memory	−0.27(1.70)	1.53(1.36)	−3.252	0.010**	−1.52(2.14)	0.38(1.96)	−2.808	0.019*
Information processing speed	0.76(2.73)	1.42(3.30)	−1.175	0.270	−1.52(3.14)	−0.47(2.96)	−2.131	0.059
Executive function	0.80(3.11)	1.64(3.85)	−1.323	0.218	−2.17(2.05)	−0.05(2.86)	−2.987	0.014*
Visuospatial function	0.71(1.56)	0.18(1.96)	0.801	0.444	−0.80(1.89)	−0.01(1.35)	−1.235	0.245

Data are presented as mean (standard deviation, SD). SCD, subjective cognitive decline; aMCI, amnestic mild cognitive impairment. *Significant differences were found between before rTMS and after rTMS. *p ≤ 0.05, **p ≤ 0.01, ***p ≤ 0.001. Most *p*-values were obtained using paired *T*-tests, except for gender (chi-square test). This study utilized the composite *Z* scores to determine the level of each cognitive domain.

### 3.2 Group ICA and ROIs

**Figure 1:**
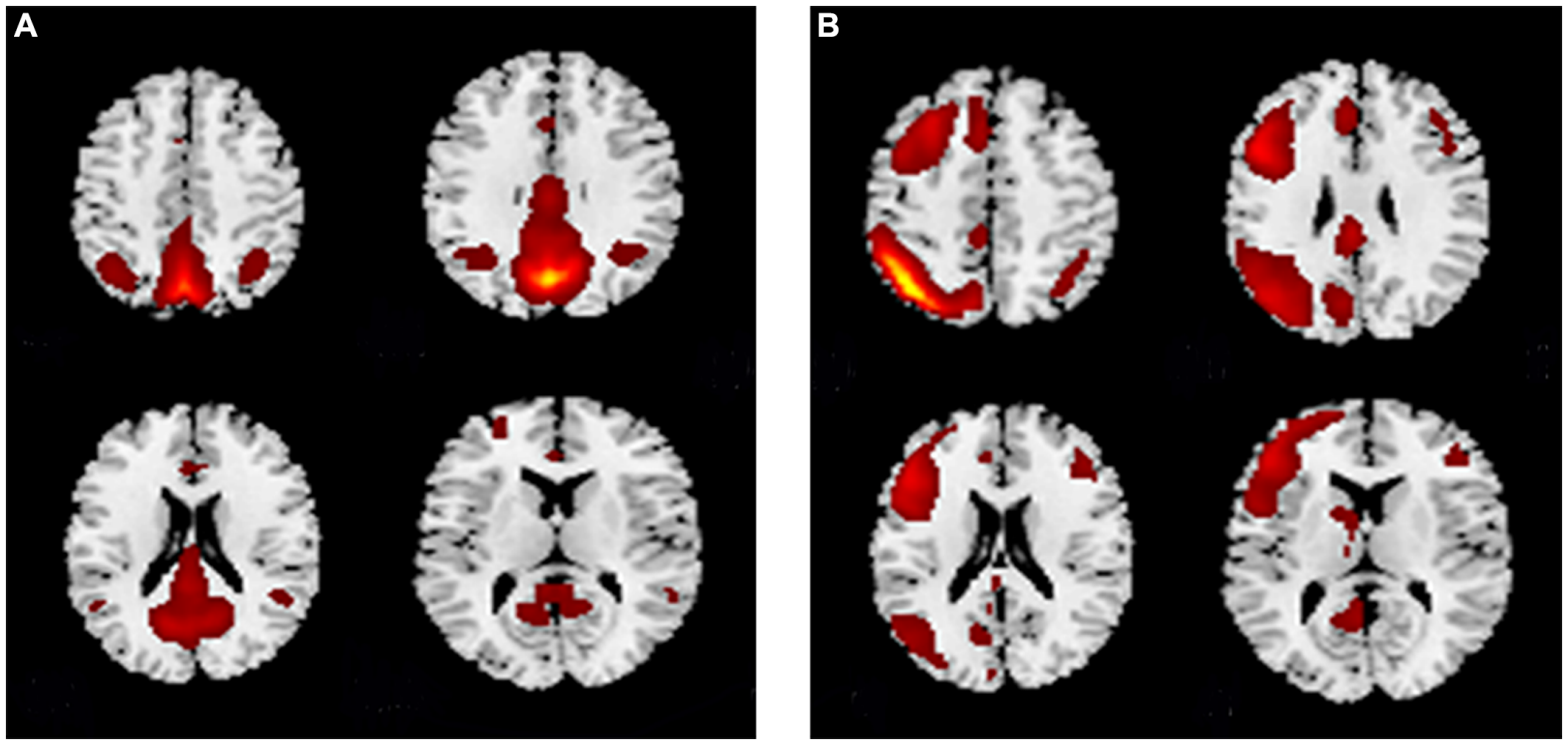
The spatial pattern of the **(A)** DMN and **(B)** CEN. **(A)** The spatial pattern of the DMN. **(B)** The spatial pattern of the CEN. DMN, default mode network; CEN, central executive network.

**Figure 2:**
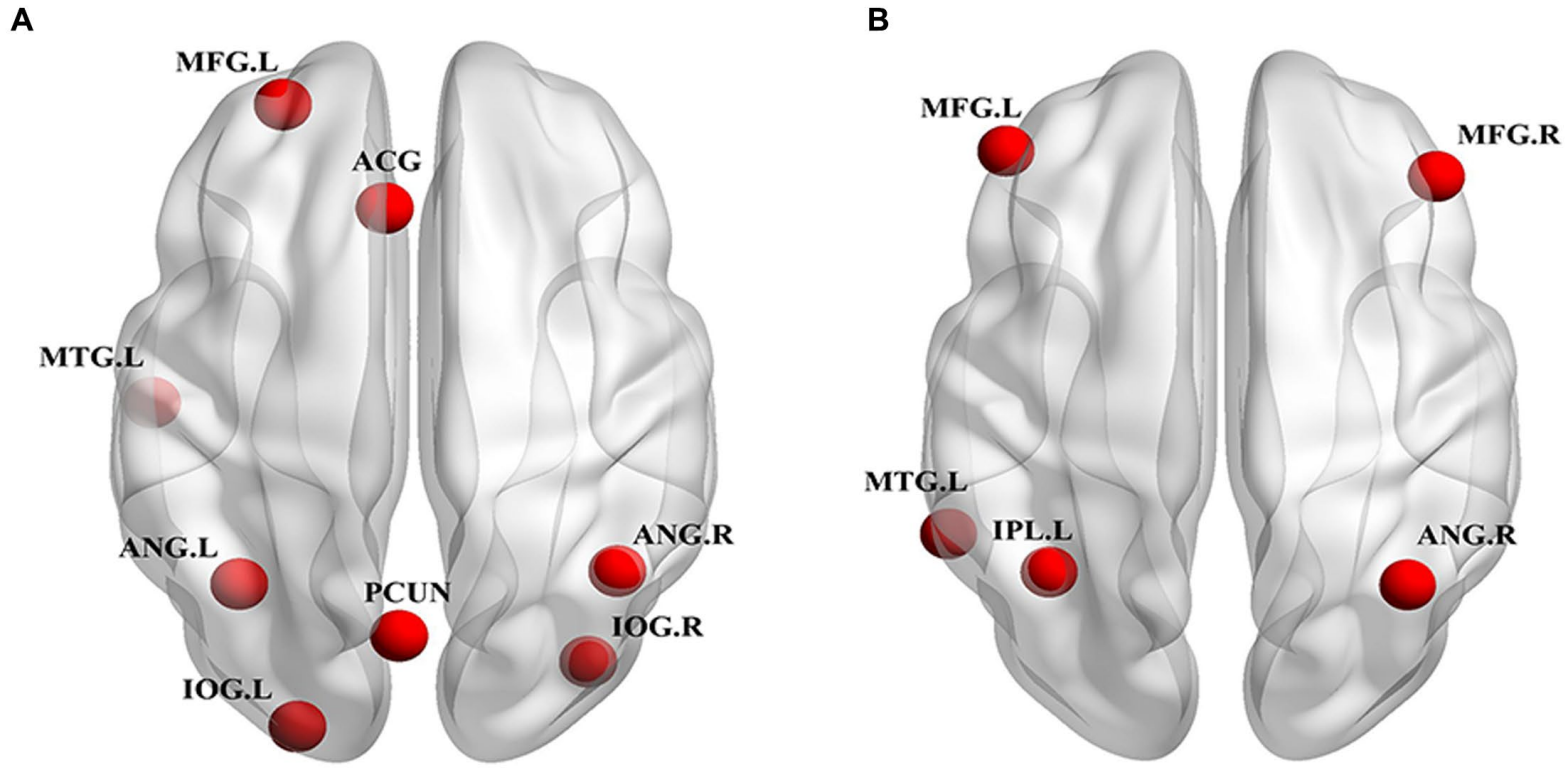
Spatial location of ROIs for DCM analyses. **(A)** DMN ROIs: PCUN, bilateral ANG, ACG, MFG.L, MTG.L, bilateral IOG. **(B)** CEN ROIs: ANG.R, IPL.L, MTG.L, bilateral MFG. ROI, region of interest; DCM, dynamic causal model; DMN, default mode network; CEN, central executive network; PCUN, precuneus; ANG, angular gyrus; ACG, anterior cingulate gyrus; MFG, middle frontal gyrus; MTG, middle temporal gyrus; IOG, inferior occipital gyrus; ANG, angular gyrus; IPL, inferior parietal lobule; L, left; R, right.

Among the 27 independent components, the 25th and 18th components exhibited the highest correlations with the DMN and CEN brain network templates, respectively. Figure 1A shows the spatial pattern of the DMN and Figure 1B shows the spatial pattern of the CEN. The whole-brain detail maps of DMN and CEN are presented in Supplementary Figure S1. The coordinates of the ROIs of the DMN and CEN, determined using the XjView toolbox, are shown in Table 3 and spatial locations are shown in Figure 2.

**Table 3:** ROIs for DCM analyses.

Anatomical region	MNI coordinate		
	*X*	*Y*	*Z*
Default mode network			
PCUN	−3	−72	30
ANG.L	−36	−60	39
ANG.R	42	−57	42
ACG	−6	27	27
MFG.L	−27	51	9
MTG.L	−54	−18	−18
IOG.L	−24	−93	−9
IOG.R	36	−78	−18
Central executive network			
ANG.R	39	−60	45
IPL.L	−36	−57	42
MTG.L	−57	−48	−15
MFG.L	−45	42	3
MFG.R	45	36	21

MNI, Montreal neurological institute; PCUN, precuneus; ANG, angular gyrus; ACG, anterior cingulate gyrus; MFG, middle frontal gyrus; MTG, middle temporal gyrus; IOG, inferior occipital gyrus; IPL, inferior parietal lobule; L, left; R, right.

### 3.3 Effective connectivity patterns of three groups

**Figure 3:**
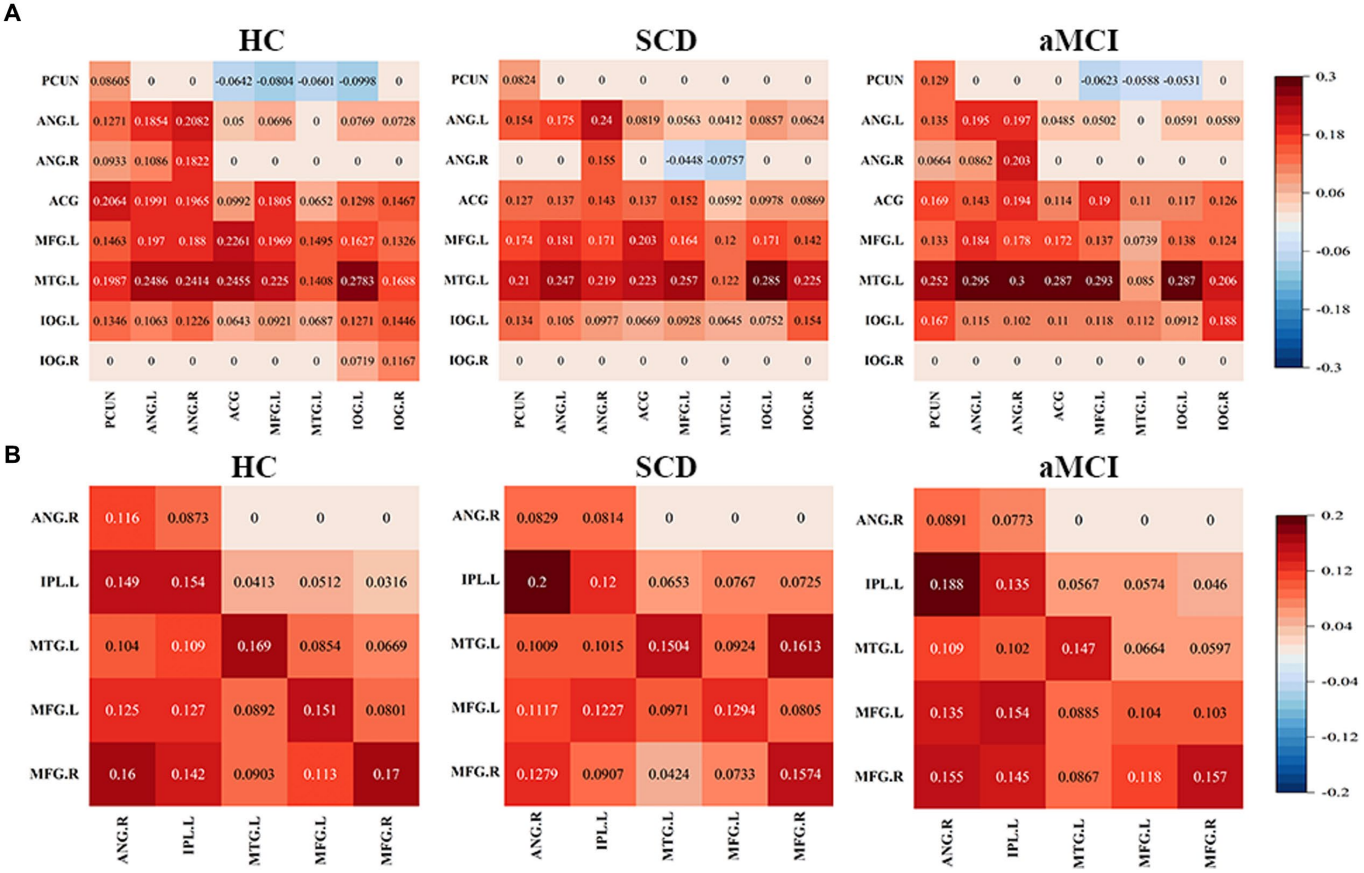
Mean values in effective connectivity for HC, patients with SCD and aMCI. **(A)** DMN effective connectivity patterns of HCs, SCD and aMCI. **(B)** CEN effective connectivity patterns of HCs, SCD and aMCI. HC, healthy controls; SCD, subjective cognitive decline; aMCI, amnestic mild cognitive impairment; DMN, default mode network; CEN, central executive network; PCUN, precuneus; ANG, angular gyrus; ACG, anterior cingulate gyrus; MFG, middle frontal gyrus; MTG, middle temporal gyrus; IOG, inferior occipital gyrus; IPL, inferior parietal lobule; L, left; R, right.

The effective connectivity patterns for the DMN and CEN for the three groups obtained through a one-sample *t*-test based on the effective connectivity strength values are shown in Figure 3. The DMN and CEN of all three groups exhibited varying degrees of excitatory or inhibitory connectivity within their respective networks.

### 3.4 Differences in effective connectivity of three groups

**Figure 4:**
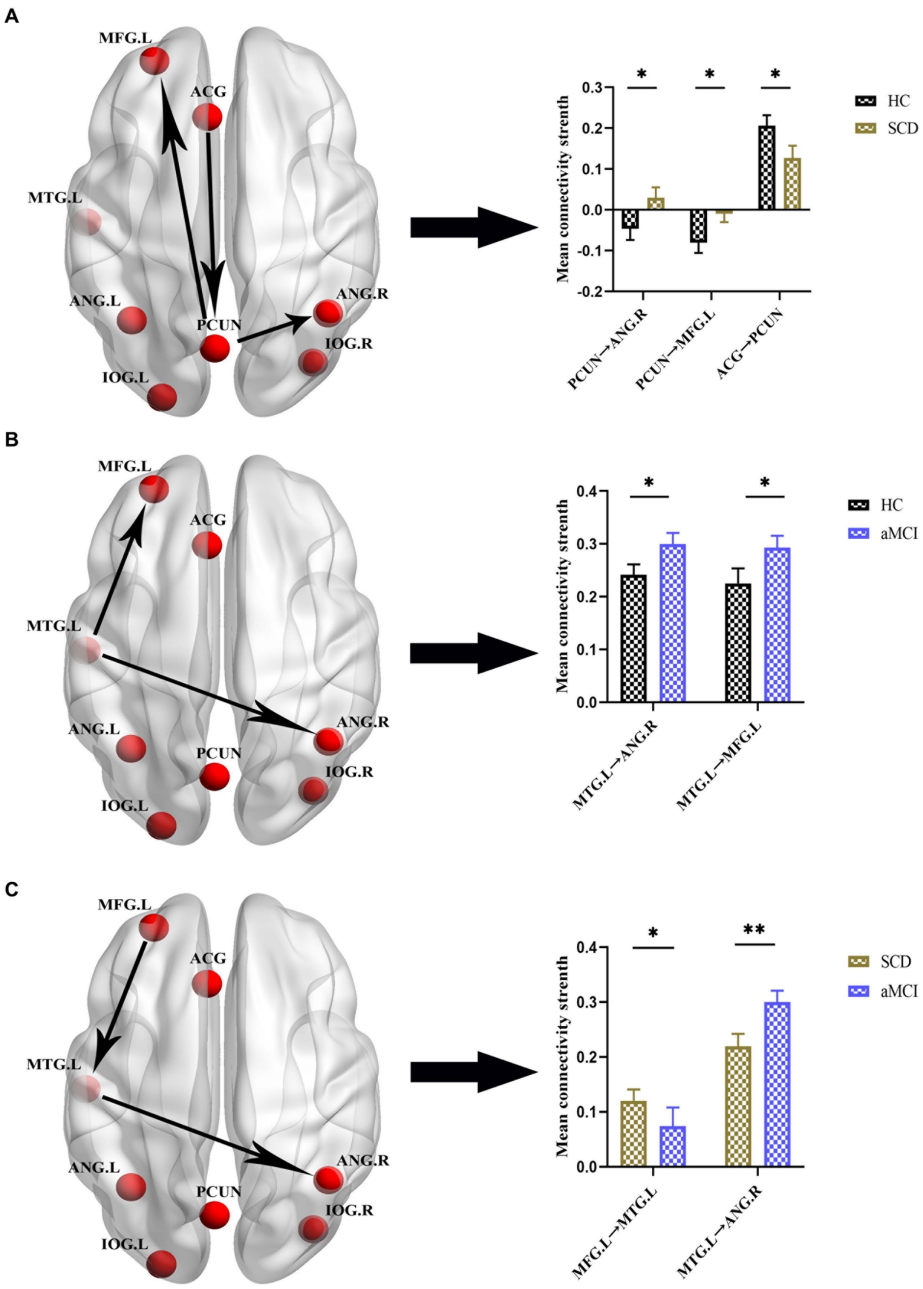
Group differences in effective connectivity HC, patients with SCD, and aMCI in DMN. **(A)** Differences in effective connectivity in patients with SCD compared to HC. A bar chart indicating the quantitative comparison of effective connectivity between these regions. **(B)** Differences in effective connectivity in patients with aMCI compared to HC. A bar chart indicating the quantitative comparison of effective connectivity between these regions. **(C)** Differences in effective connectivity in patients with aMCI compared to SCD. A bar chart indicating the quantitative comparison of effective connectivity between these regions. *Significant different (*p ≤ 0.05, **p ≤ 0.01, ***p ≤ 0.001); error bar, standard error of the mean (SEM). HC, healthy controls; SCD, subjective cognitive decline; aMCI, amnestic mild cognitive impairment; DMN, default mode network; PCUN, precuneus; ANG, angular gyrus; ACG, anterior cingulate gyrus; MFG, middle frontal gyrus; MTG, middle temporal gyrus; L, left; R, right.

**Figure 5:**
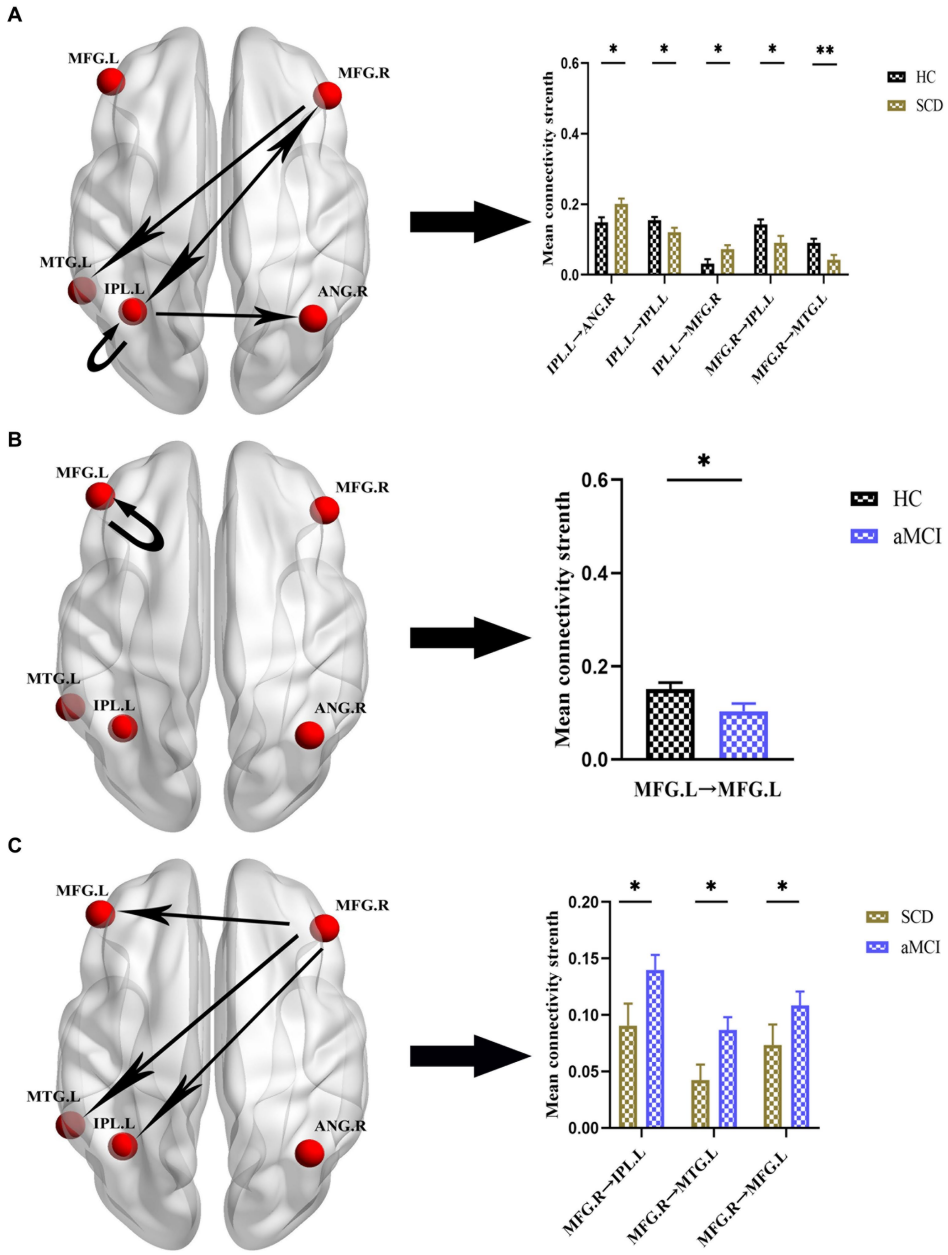
Group differences in effective connectivity HC, patients with SCD, and aMCI in CEN. **(A)** Differences in effective connectivity in patients with SCD compared to HC. A bar chart indicating the quantitative comparison of effective connectivity between these regions. **(B)** Differences in effective connectivity in patients with aMCI compared to HC. A bar chart indicating the quantitative comparison of effective connectivity between these regions. **(C)** Differences in effective connectivity in patients with aMCI compared to SCD. A bar chart indicating the quantitative comparison of effective connectivity between these regions. *Significant different (*p ≤ 0.05, **p ≤ 0.01, ***p ≤ 0.001); error bar, standard error of the mean (SEM). HC, healthy controls; SCD, subjective cognitive decline; aMCI, amnestic mild cognitive impairment; CEN, central executive network; IPL, inferior parietal lobule; ANG, angular gyrus; MFG, middle frontal gyrus; MTG, middle temporal gyrus; L, left; R, right.

Figures 4, 5 and Table 4 show the differences in effective connectivity among the three groups. In the DMN, compared to the HC group, the inhibitory connectivity from PCUN to ANG.R switched to excitatory connectivity, the inhibitory connectivity from PCUN to MFG.L decreased, and the excitatory connectivity from ACG to PCUN decreased in the SCD group. Meanwhile, excitatory connectivity from MTG.L to ANG.R and MFG.L increased in the aMCI group. Compared to the SCD group, the excitatory connectivity from MFG. L to MTG.L decreased, while that from MTG.L to ANG.R increased in the aMCI group. In the CEN, compared to the HC group, excitatory connectivity from IPL.L to ANG.R and MFG.R increased in the SCD group, while that from IPL.L to IPL.L decreased, and excitatory connectivity from MFG.R to IPL.L and MTG.L decreased. Conversely, in the aMCI group, excitatory connectivity from MFG.L to MFG.L decreased in the CEN. Compared to the SCD group, the aMCI group showed increased excitatory connectivity from MFG.R to IPL.L, MTG.L, and MFG.L.

**Table 4:** Effective connectivity parameters differences across three groups.

Brain regions	Mean strength		*F*-values
**Default mode network**			
**HC vs. SCD**	HC	SCD	
PCUN→ANG.R	−0.0462 ± 0.2565	0.0294 ± 0.2187	0.044
PCUN→MFG.L	−0.0804 ± 0.2328	−0.0097 ± 0.1729	0.035
ACG → PCUN	0.2064 ± 0.2360	0.1271 ± 0.2566	0.036
**HC vs. aMCI**	HC	aMCI	
MTG.L → ANG.R	0.2414 ± 0.1837	0.3000 ± 0.1932	0.045
MTG.L → MFG.L	0.2250 ± 0.2654	0.2933 ± 0.2065	0.045
**SCD vs. aMCI**	SCD	aMCI	
MFG.L → MTG.L	0.1203 ± 0.1749	0.0739 ± 0.3164	0.033
MTG.L → ANG.R	0.2190 ± 0.1952	0.3000 ± 0.1932	0.008
**Central executive network**			
**HC vs. SCD**	HC	SCD	
IPL.L → ANG.R	0.1488 ± 0.1312	0.2014 ± 0.1258	0.012
IPL.L → IPL.L	0.1540 ± 0.0884	0.1200 ± 0.1134	0.035
IPL.L → MFG.R	0.0316 ± 0.1162	0.0725 ± 0.0975	0.017
MFG.R → IPL.L	0.1424 ± 0.1342	0.0907 ± 0.1653	0.026
MFG.R → MTG.L	0.0903 ± 0.1085	0.0424 ± 0.1163	0.007
**HC vs. aMCI**	HC	aMCI	
MFG.L → MFG.L	0.1509 ± 0.1301	0.1038 ± 0.1514	0.028
**SCD vs. aMCI**	SCD	aMCI	
MFG.R → IPL.L	0.0907 ± 0.1653	0.1446 ± 0.1357	0.020
MFG.R → MTG.L	0.0424 ± 0.1163	0.0867 ± 0.1063	0.012
MFG.R → MFG.L	0.0733 ± 0.1552	0.1177 ± 0.1346	0.027

Data are presented as mean (standard deviation, SD). HC, healthy controls; SCD, subjective cognitive decline; aMCI, amnestic mild cognitive impairment; PCUN, precuneus; ANG, angular gyrus; MFG, middle frontal gyrus; ACG, anterior cingulate gyrus; MTG, middle temporal gyrus; IPL, inferior parietal lobule; L, left; R, right.

### 3.5 Change of effective connectivity after rTMS

**Figure 6:**
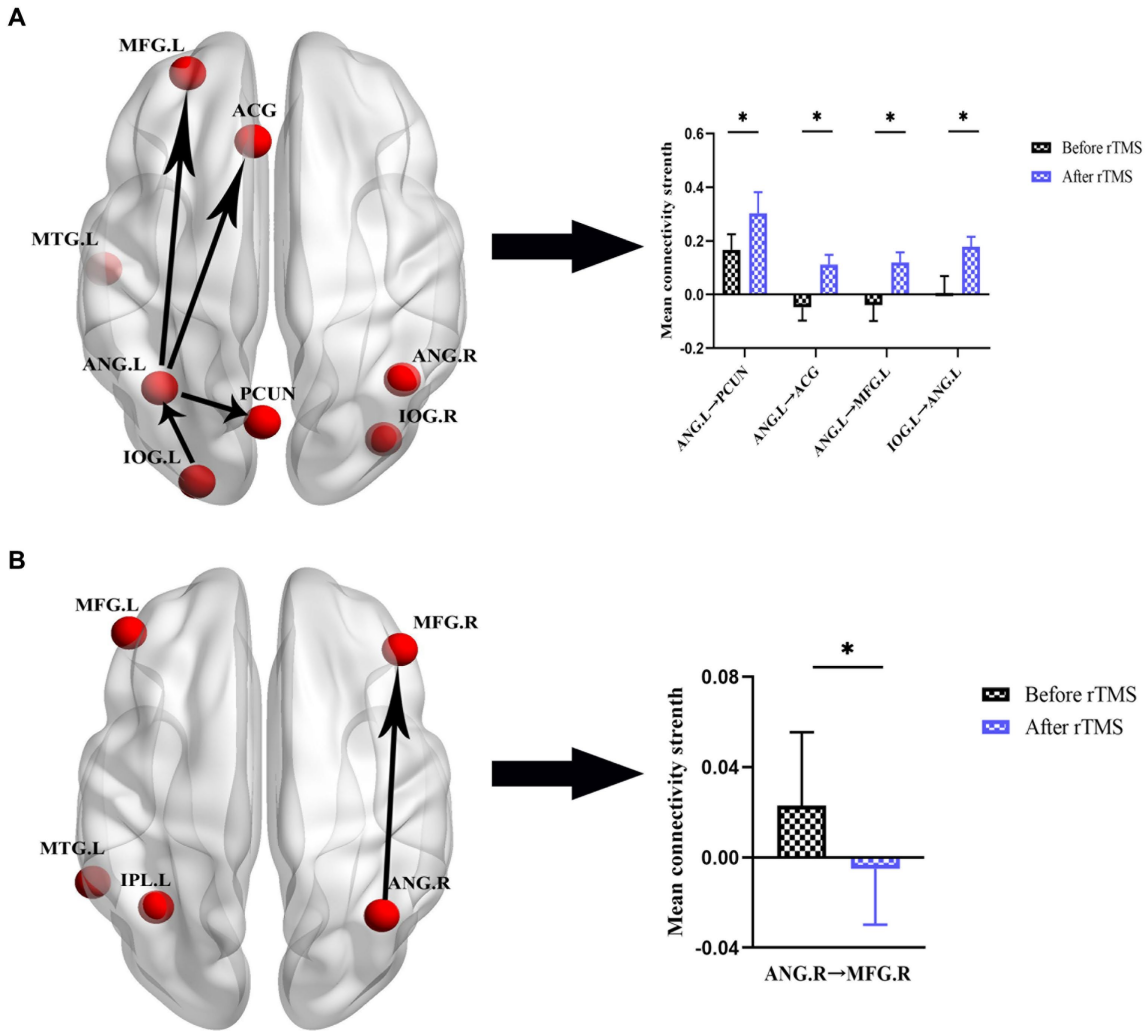
Effective connectivity changes in DMN and CEN before and after rTMS in patients with SCD. **(A)** Effective connectivity changes in DMN. A bar chart indicating the quantitative comparison of effective connectivity between these regions. **(B)** Effective connectivity changes in CEN. A bar chart indicating the quantitative comparison of effective connectivity between these regions. *Significant different (*p ≤ 0.05, **p ≤ 0.01, ***p ≤ 0.001); error bar, standard error of the mean (SEM). DMN, default mode network; CEN, central executive network; SCD, subjective cognitive decline; rTMS, repetitive transcranial magnetic stimulation; ANG, angular gyrus; PCUN, precuneus; ACG, anterior cingulate gyrus; MFG, middle frontal gyrus; IOG, inferior occipital gyrus; L, left; R, right.

After the rTMS intervention, notable alterations in effective connectivity were observed. In the SCD group, within the DMN network, excitatory connectivity from ANG.L to PCUN was elevated, and inhibitory connectivity from ANG.L to ACG, MFG.L, and IOG.L to ANG.L was transformed into excitatory connectivity. Within the CEN network, the excitatory connectivity from ANG.R to MFG. R was transformed into inhibitory connectivity. However, we did not observe significant changes in effective connectivity in the aMCI group. The specific changes in effective connectivity are shown in Figure 6 and Table 5.

**Table 5:** Effective connectivity parameters change of SCD and aMCI before rTMS and after rTMS.

Brain regions	Mean strength		*T*-values
**Default mode network**			
**SCD**	Before	After	
ANG.L → PCUN	0.1667 ± 0.1838	0.3033 ± 0.2471	0.044
ANG.L → ACG	−0.0479 ± 0.1560	0.1123 ± 0.1148	0.035
ANG.L → MFG.L	−0.0393 ± 0.1895	0.1202 ± 0.1195	0.034
IOG.L → ANG.L	−0.0220 ± 0.2295	0.1782 ± 0.1183	0.029
**aMCI**			
None			
**Central executive network**			
**SCD**	Before	After	
ANG.R → MFG.R	0.0230 ± 0.1025	−0.0300 ± 0.0829	0.031
**aMCI**			
None			

Data are presented as mean (standard deviation, SD). SCD, subjective cognitive decline; aMCI, amnestic mild cognitive impairment; ANG, angular gyrus; PCUN, precuneus; ACG, anterior cingulate gyrus; MFG, middle frontal gyrus; IOG, inferior occipital gyrus; L, left; R, right.

### 3.6 Correlation analysis with neuropsychological scores

**Figure 7:**
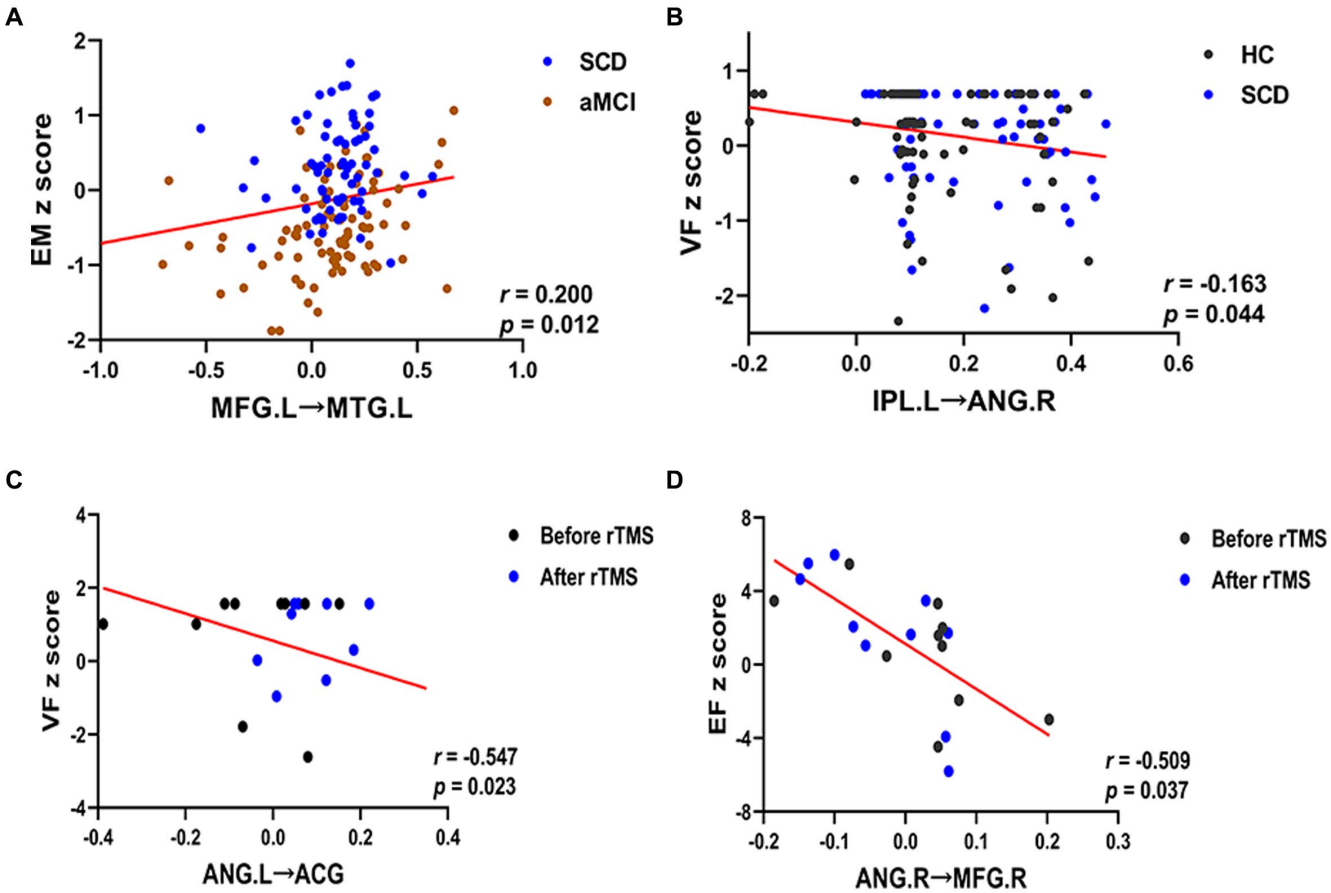
Relationship between altered effective connectivity and cognitive function in HC, SCD, aMCI, before rTMS and after rTMS. **(A)** Relationship between effective connectivity of the MFG.L to MTG.L and episodic memory in patients with SCD and aMCI in the DMN. **(B)** Relationship between effective connectivity of the IPL.L to ANG.R and visuospatial function in patients with HC and SCD in the CEN. **(C)** Relationship between effective connectivity of the ANG.L to ACG and visuospatial function in patients with the SCD before and after rTMS in the DMN. **(D)** Relationship between effective connectivity of the ANG.R to MFG.R and executive function in patients with the SCD before and after rTMS in the CEN. HC, healthy controls; SCD, subjective cognitive decline; aMCI, amnestic mild cognitive impairment; rTMS, repetitive transcranial magnetic stimulation; DMN, default mode network; CEN, central executive network; EM, episodic memory; VF, visuospatial function; EF, executive function. MFG, middle frontal gyrus; MTG, middle temporal gyrus; IPL, inferior parietal lobule; ANG, angular gyrus; ACG, anterior cingulate gyrus; L, left; R, right.

Correlation analyses were performed to explore the associations between regions with significantly altered effective connectivity and cognitive domains (*p* < 0.05), and age, gender, and educational level were considered as covariates. The results showed that in both SCD and aMCI groups, the effective connectivity between the DMN networks MFG.L and MTG.L was positively correlated with episodic memory (*r* = 0.200, *p* = 0.012). Moreover, the effective connectivity of the CEN network IPL.L to ANG.R was negatively correlated with visuospatial function in the HC and SCD groups (*r* = −0.163, *p* = 0.044). After the rTMS intervention, the effective connectivity from ANG.L to ACG in the DMN network was negatively correlated with visuospatial function in the SCD group (*r* = −0.547, *p* = 0.023). Meanwhile, the effective connectivity from ANG.R to MFG.R in the CEN network was negatively correlated with executive function in the SCD group (*r* = −0.509, *p* = 0.037; Figure 7).

## 4 Discussion

This study aimed to investigate the regulation and effect of PCUN-targeted rTMS intervention on effective connectivity and cognitive function within brain networks. Our investigation presents two main findings. First, we found that within the DMN and CEN, the spectrum of preclinical AD had different causal patterns, indicating effective connectivity within the brain networks associated with various pathological states in the spectrum of preclinical AD. Secondly, after the rTMS intervention, the spectrum of preclinical AD showed changes in effective connectivity, accompanied by improvements in cognitive function. Therefore, this study showed that the rTMS intervention targeting the PCUN can modulate the effective connectivity patterns of the brain network in the spectrum of preclinical AD and improve cognitive function. Thus, the PCUN can be considered an ideal target for rTMS intervention.

### 4.1 Altered DMN and CEN effective connectivity patterns in patients with SCD and aMCI

The DMN is considered the first large-scale system to be compromised in the progression of AD. Studies on the strength of FC between important nodes of DMN in patients with SCD showed de-coupling of DMN nodes (Dillen et al., 2017). In addition, DMN, a key hub in the spectrum of preclinical AD, is thought to be more susceptible to amyloid-beta deposition and glucose hypometabolism (Mutlu et al., 2017). Using fMRI and diffusion-weighted imaging (DWI), Zhou et al. found abnormal structural and functional connectivity within the DMN in patients with MCI, demonstrating a significant association with cognitive decline (Zhou et al., 2022). The above studies indicate that in the spectrum of preclinical AD, the DMN exhibits altered functional, structural, and metabolic changes. Consistent with these findings, the present study stated that the effective connectivity pattern of the DMN is altered in the spectrum of preclinical AD. In patients with SCD, we observed a decrease in effective connectivity from the ACG to the PCUN and an increase in effective connectivity from the PCUN to the ANG.R and MFG.L. This suggests that during the SCD phase, the PCUN, a core node of the DMN, can compensate for the increased activity to maintain normal levels of cognition despite the presence of partially impaired brain area function. Similarly, in patients with MCI, the effective connectivity from MTG.L to ANG.R and MFG.L was elevated. This is consistent with a recent study that highlights increased neural activity in the frontotemporal region as the disease progresses to resist neurotoxic effects (Gour et al., 2011). We also found that the effective connectivity from MFG.L to MTG.L decreased was positively correlated with the decline in memory in the SCD and aMCI groups. This suggests that MFG. L is damaged during the disease process and is closely related to cognitive abilities, and the degree of damage may predict the decline in memory. Therefore, altered effective connectivity patterns observed in the DMN in the spectrum of preclinical AD can be considered important neuroimaging evidence.

Neuroimaging studies have shown the existence of the brain network degeneration hypothesis in the AD spectrum (Mutlu et al., 2017). An arterial spin labeling perfusion and fMRI study confirmed that decreases in CBF and amplitude low-frequency fluctuation (ALFF) indicate disruption of the CEN (Zheng et al., 2019). Liao et al. demonstrated abnormal interhemispheric CEN network connectivity in patients with MCI using voxel-mirrored homotopic connectivity (Liao et al., 2018). In addition, Granger causality analysis confirmed that progressive MCI cases exhibit different patterns of effective CEN connectivity, potentially serving as a biomarker for predicting the progression of AD (Cai et al., 2017). Similarly, this study used spDCM to demonstrate altered effective connectivity patterns in the CEN in patients with SCD and aMCI. In patients with SCD, effective connectivity from IPL.L to ANG was increased and that to IPL.L and MFG.R was decreased. In contrast, patients with aMCI showed decreased self-connection within MFG.L. This is consistent with the study by Wu et al. (2014), which showed a concurrent decrease in the functional connectivity of the CEN. Decreased effective connectivity is suggestive of damage to brain regions, whereas increased effective connectivity may reflect a maladaptive compensatory mechanism. This implies a transition from a healthy neuroplastic compensation mechanism to maladaptive processes within damaged brain regions, triggered by factors such as GM atrophy and hypoperfusion (Mesulam, 2006). The research findings suggest that IPL, as a multimodal and heterogeneous brain region, plays a crucial role in the CEN (Uddin et al., 2011). Therefore, based on our partial correlation results, it can be inferred that the increased effective connectivity of IPL reflects its compensatory mechanism. However, this mechanism may vary in its impact on different brain networks, thereby exhibiting a negative association with visual function. Elevated and decreased within-network effective connectivity in the DMN and CEN networks have been observed in both SCD and aMCI patients, possibly reflecting complex brain regulation following cognitive impairment. This may involve intricate interactions within and between multiple brain networks, warranting further in-depth investigations in future research. These insights offer novel perspectives on the pathogenesis and neuroimaging of the spectrum of preclinical AD.

### 4.2 Changes in effective connectivity patterns and cognition after rTMS in patients with SCD

After undergoing two courses of rTMS treatment, notable changes were observed in the effective connectivity patterns of the DMN and CEN in patients with SCD, including improvements in episodic memory. Specifically, the effective connectivity of the ANG.L to the PCUN, ACG, MFG.L, and IOG.L in the DMN increased, and the previously excitatory effective connectivity from ANG.R to MFG.R in the CEN transformed into inhibitory connectivity. ANG plays an important role in the development, progression, and transition of the spectrum of AD (Hu et al., 2022). Cai et al. (2020) documented a decrease in ANG FC in patients with SCD. Furthermore, decreased CBF in ANG was observed in patients with MCI who eventually progressed to AD (Hirao et al., 2005). IOG is mainly related to vision, and a study reported an increase in fALFF values in patients with SCD (Sun et al., 2016). It has been suggested that local stimulation of rTMS acting on a target site can be transmitted via synapses to interconnected nodes, facilitating regulation within brain regions and networks (Chen et al., 2021). Anatomical and functional studies on the PCUN have shown that is a complex cortical and subcortical structure characterized by extensive connectivity (Cavanna and Trimble, 2006). Therefore, we hypothesize that local stimulation of rTMS targeting the PCUN is transmitted synaptically to susceptible brain regions connected to it, thereby modulating the DMN and CEN networks and causing changes in effective connectivity patterns.

A previous meta-analysis has shown that episodic memory requires the involvement of multi-brain networks (Liang et al., 2022). DMN and CEN, as the two core networks for maintaining cognition, also play an important role in episodic memory. A long-term memory model has shown that the PCUN is involved in both the encoding and retrieval of episodic memory (Daselaar, 2009). PCUN-targeted rTMS improves episodic memory (Chen et al., 2021). The ANG is widely recognized as a pivotal interface, playing a crucial role in the processes of episodic simulation and episodic memory (Thakral et al., 2017). The application of facilitatory TMS to the ANG has been linked to improvements in episodic memory performance (Nilakantan et al., 2017). Furthermore, the IOG predominantly processes visual information and is closely associated with visuospatial and executive functions (Bilo et al., 2013). Cognitive-behavioral investigations have provided evidence highlighting the significant contribution of visual attention in the retrieval of episodic memories (Guerin et al., 2012). According to the results of partial correlation analysis, alterations in effective connectivity of the ANG were found to exhibit a negative correlation with visuospatial and executive functions. This finding suggests the presence of a complex regulatory mechanism involving interplay among various brain regions, which may contribute to the suppression or disruption of visuospatial and executive functions, while not necessarily indicating a significant decline in functionality. Thus, we propose that in patients with SCD, PCUN-targeted rTMS regulates the effective connectivity patterns within the DMN and CEN networks. This modulation activates brain regions integral to episodic memory function and promotes functional integration, thereby improving episodic memory. In summary, we believe that SCD is an ideal stage for rTMS interventions aimed to improve cognition by regulating effective connectivity patterns.

### 4.3 Cognitive changes after rTMS intervention in patients with aMCI

The present study showed noteworthy improvements in episodic memory and executive function in patients with aMCI after PCUN-targeted rTMS. However, we did not observe changes in the effective connectivity patterns of the DMN and CEN. Episodic memory is the ability to encode, retain, and retrieve information related to personal events and experiences that occurred at a specific time and place. Decreased episodic memory function is a central feature of aMCI (Bai et al., 2016). Meanwhile, executive functions include complex attention, working memory, verbal and visual organization, planning, sound judgment, and reasoning capacities. Executive dysfunction has been shown to predict the progression of aMCI to AD (Tabert et al., 2002).

A study using high-frequency rTMS showed sustained improvements in episodic memory in patients with MCI (Drumond Marra et al., 2015). Some studies have also shown the potential of rTMS to improve attention and psychomotor speed in patients with MCI and AD (Anderkova et al., 2015). A recent systematic review including studies on rTMS intervention in patients with MCI and AD showed that rTMS can improve and restore impaired cognitive function (Chou et al., 2020). These findings are consistent with the current study, which showed improved cognitive function in patients with aMCI after rTMS treatment.

However, unlike patients with SCD, we did not observe any changes in the effective connectivity patterns of the DMN and CEN in patients with aMCI. There may be a possibility that the differential response to rTMS intervention in SCD and aMCI patients could be attributed to the distinct baseline effective functional connectivity and varying degrees of impairment. Moreover, these discrepancies may be attributed to the differential mechanisms by which rTMS influences SCD and aMCI. Despite both being prodromal stages of Alzheimer’s disease, rTMS exerts its effects through distinct modalities in these conditions. In SCD patients, rTMS ameliorates cognitive function by modulating the effective connectivity patterns of diverse cerebral networks. Conversely, in the case of aMCI patients, rTMS may operate via alternative pathways. A previous study showed that correction of disruptions in the structure of the hippocampus and its connectivity with MTG can causally enhance episodic memory in aMCI (Chen et al., 2022). It has also been shown that rTMS can increase the expression of brain-derived neurotrophic factor (BDNF) and vascular endothelial growth factor (VEGF), thereby increasing synaptic neuroplasticity and thus improving cognition (Zhang et al., 2015). Cristina et al. proposed that rTMS improves cognition by promoting the compensatory recruitment potential of other neural networks (Solé-Padullés et al., 2006). Therefore, we propose that rTMS is a promising non-invasive technique for enhancing cognitive function in SCD and aMCI patients. Specifically, rTMS achieves this by modulating the effective connectivity patterns of cerebral networks in SCD patients, while the mechanisms underlying its effects on aMCI patients remain to be further investigated in future studies. Based on our findings, we propose that rTMS can better improve cognitive function in patients with SCD by modulating effective connectivity within the brain network.

### 4.4 Limitations

The main limitation of this study is the relatively small sample size. However, to ensure the accuracy of the experiment, all subjects met the diagnostic criteria and were strictly grouped. In addition, we should establish a more comprehensive definition of the preclinical spectrum of AD to facilitate more extensive research (Zhou, 2021). Furthermore, this study currently lacks a sham rTMS group for control comparison. However, we are actively recruiting more participants and utilizing a more advanced stereotactic neuro-navigation system to monitor coil positioning for target localization. This will enable us to conduct more comprehensive and in-depth research in the future, and further advancing our understanding in this area. Despite these limitations, to our knowledge, this is the first study to target the efficacy of rTMS at the level of effective connectivity in brain networks, and we once again demonstrate the effectiveness of rTMS therapy targeting PCUN.

## 5 Conclusion

This study demonstrates that SCD and aMCI are changed in the effective connectivity patterns of DMN and CEN as the spectrum of preclinical AD. In addition, after two courses of PCUN-targeted rTMS treatment, the effective connectivity of the spectrum of preclinical AD was modulated, accompanied by cognitive improvement. These results prove that PCUN can be used as an effective target for rTMS, acting on the spectrum of preclinical AD, especially in the SCD stage, which can delay or reverse the disease process. This provides new insights into the pathogenesis and clinical treatment of the spectrum of preclinical AD.

## Data availability statement

The raw data supporting the conclusions of this article will be made available by the authors, without undue reservation.

## Ethics statement

The studies involving humans were approved by the Human Participants Ethics Committee of the Affiliated Brain Hospital of Nanjing Medical University (Nos. 2018-KY010-01 and 2020-KY010-02). Written informed consent was obtained from all subjects prior to enrolment. The studies were conducted in accordance with the local legislation and institutional requirements. The participants provided their written informed consent to participate in this study.

## Author contributions

XuL: Formal analysis, Methodology, Writing – original draft. CXu: Methodology, Writing – original draft. DZ: Data curation, Methodology, Writing – original draft. QY: Data curation, Writing – original draft. WQ: Formal analysis, Writing – original draft. YR: Investigation, Writing – original draft. SC: Software, Writing – original draft. YS: Validation, Writing – original draft. HW: Data curation, Writing – original draft. XiL: Funding acquisition, Writing – original draft. CXi: Project administration, Writing – review & editing. JC: Funding acquisition, Project administration, Resources, Writing – review & editing.

## Footnotes

^1^http://icatb.sourceforge.net/

^2^http://www.alivelearn.net/xjview

^3^http://www.fil.ion.ucl.ac.uk/spm/software/spm12/
