# Supplementary material for: Repetitive transcranial magnetic stimulation regulates effective connectivity patterns of brain networks in the spectrum of preclinical Alzheimer’s disease
Source: Front Aging Neurosci. 2024 Feb 12;16:1343926. doi: 10.3389/fnagi.2024.1343926 (PMC10894951; doi:10.3389/fnagi.2024.1343926)
Supplement: Supplementary file 1 [file Data_Sheet_1.doc]

**Supporting Information**

### Supplementary Method

### S.1 NBH-ADsnp database

Data used in this study were obtained from the Nanjing Brain Hospital-Alzheimer’s Disease Spectrum Neuroimaging Project Version 2 (NBH-ADsnp-2) database. NBH-ADsnp-2 is an upgrade of NBH-ADsnp and is derived from an Alzheimer’s Disease Spectrum Neuroimaging cooperative Project that was jointly built in September 2022 by Department of Radiology, Nanjing Drum Tower Hospital and Department of Radiology and Neurology, the Affiliated Brain Hospital of Nanjing Medical University. Prof. Jiu Chen, PhD, MD, from Nanjing Drum Tower Hospital, and Xingjian Lin, MD and Chaoyong Xiao, MD, from the Affiliated Brain Hospital of Nanjing Medical University, acts as the principal investigator of NBH-ADsnp-2. NBH-ADsnp-2 was initiated by Dr. Jiu Chen, Dr. Xingjian Lin, and Dr. Chaoyong Xiao and was named by Dr. Chen, Dr. Lin, Dr. Xiao's cooperative research group (discussed by Chen Xue, Guan-jie Hu, Wen-wen Xu, Wan Liu, Wen-zhang Qi, Si-yu Wang, Jia-ni Xu, Shan-shan Chen, Honglin Ge, Zheng Yan, Yu Song, Qianqian Yuan, Huimin Wu, Xuhong Liang, Xinyi Yang and finally verified by Jiu Chen, Xingjian Lin, and Chaoyong Xiao). NBH-ADsnp-2 is an observational and intervention study which includes cross-sectional and longitudinal follow-up components. The goal of NBH-ADsnp-2 is to identify early neuroimaging biomarkers of preclinical Alzheimer’s Disease (AD) spectrum {subjective cognitive decline (SCD), amnestic mild cognitive impairment (aMCI), amnestic mild cognitive impairment (naMCI), and AD}, to predict the disease progression of individuals within preclinical AD spectrum, and to provide imaging-based targets for individualized intervention in order to prevent the disease deterioration from preclinical stages to the eventually progressed AD. All subjects in NBH-ADsnp-2, who were all Han Chinese and right-handed, were recruited initially from hospitals and local communities by advertising and by means of broadcasting. This database used a standardized clinical evaluation protocol that included a medical history interview, neurologic examination, a battery of neurocognitive assessment, and resting-state MRI scan (T1, T2, 3D T1, DTI, and BOLD) for all participants (healthy controls, SCD, naMCI, aMCI, and AD). In addition, MRI data collected after 2 and 4 weeks of rTMS intervention in patients with SCD and MCI were added after the database upgrade. All subjects and their study partners completed the informed consent process, and the study protocols were reviewed and approved by the responsible Human Participants Ethics Committee of the Affiliated Brain Hospital of Nanjing Medical University (No. 2018-KY010-01, No. 2020-KY010-02, No.2021-KY029-01, No. 2021-KY009-01, No. 2022-KY042-01, No. ChiCTR2000034533, No. ChiCTR1900022287).

The general eligibility, inclusion, and exclusion criteria for NBH-ADsnp-2 subjects can be found as follows:

Inclusion criteria of SCD subjects were identified meeting the published SCD research criteria proposed by the Subjective Cognitive Decline Initiative (SCD-I)(Jesse*n et a*l. 2014), and the detailed inclusion criteria have been described in our previously published studies(Xu*e et a*l. 2019), as follows: (a) self-reported persistent memory decline, which was confirmed by an informant; (b) Subjective Cognitive Decline Questionnaire (SCD-Q) score > 5(Ha*o et a*l. 2017; Ya*n et a*l. 2018; Cedre*s et a*l. 2019); (c) performance within the normal range on MMSE and MoCA (adjusted for age and education); (d) Clinical Dementia Rating (CDR) = 0; and (e) subjects aged between 50 and 80 years old.

Inclusion criteria of naMCI subjects were referenced to previous studies(Dun*n et a*l. 2014; Xu*e et a*l. 2019), as follows: a) normal overall cognitive function just like aMCI; b) the tests scores about memory function are in the normal range while deficits were present on other cognitive domains including visual spatial function, executive function, language function, and information processing speed; and (c) subjects aged between 50 and 80 years old.

Inclusion criteria of aMCI subjects were identified meeting the diagnostic criteria defined by Peterson et al.(Peterse*n et a*l. 1999)as well as the revised consensus standards presented by Winblad et al.(Winbla*d et a*l. 2004), and the detailed inclusion criteria have been described in our previously published studies(Che*n et a*l. 2016, 2021, 2022; Alzheimer’s Disease Neuroimaging Initiativ*e et a*l. 2019; Xu*e et a*l. 2019), as follows: (a) memory complaint preferably corroborated by an informant or the subject for more than 3 months; (b) objective memory impairment adjusted for age and educational level; (c) normal general cognitive function of MMSE score equal or above 24; (d) no or minimal impairment in daily living activities; (e) CDR=0.5; (f) subjects aged between 50 and 80 years old; and (g) absence of dementia symptoms that were not sufficient to meet the criteria of the National Institute of Neurological and Communicative Disorders and Stroke or the AD and Related Disorders Association criteria for AD.

Inclusion criteria of CN subjects was identified meeting the following rules: (a) without memory complaint; (b) normal cognitive performance matched with age and education; (c) CDR=0; (d) MMSE ≥ 26; and (e) subjects aged between 50 and 80 years old(Xu*e et a*l. 2019; Che*n et a*l. 2020, 2021, 2022).

The detailed exclusion criteria for all subjects have been described in our previously published studies(Che*n et a*l. 2016, 2021, 2022; Alzheimer’s Disease Neuroimaging Initiativ*e et a*l. 2019; Xu*e et a*l. 2019), as follows: (a) a past history of stroke (modified Hachinski Ischemic Scale Score of > 4), alcoholism, head injury, brain tumors, Parkinson’s disease, epilepsy, encephalitis, major depression (excluded by HAMD), or other neurological or psychiatric illness (excluded by clinical assessment and case history); (b) major medical illness (e.g., cancer, anemia, thyroid dysfunction, syphilis, or HIV); (c) severe visual or hearing loss; (d) unable to complete neuropsychological tests or with a contraindication for MRI, and (5) T2-weighted MRI showing major white matter (WM) changes, infarction, or other lesions (two experienced radiologists analyzed the scans). All patients had no any medications.

### S.2 Neuropsychological assessments for the NBH-ADsnp database

Neurocognitive assessments were as described in our previously published studies. The general cognitive functions data included the MMSE, the ADL, the MDRS-2, the MoCA, the SCD-Q, the CDR, and the HAMD. The episodic memory data contained the Auditory Verbal Memory Test-20min-delayed recall (AVLT-20-min DR), the Logical Memory Test -20 min delayed recall (LMT-20-min DR), and the Rey Complex Figure Test 20min delayed recall (CFT-20-min DR). The executive function data were derived from the Category Verbal Fluency Test (VFT) (including the VFT-animals and the VFT-objects), the Digit Span Test backward (DST-backward), part B of the Trail Making Test (TMT-B), part C of the Stroop Test (Stroop C), and Semantic Similarity. The information processing speed data were obtained from the Symbol Digit Modalities Test (DSST), part A of the Trail Making Test (TMT-A), part A and B of the Stroop Test. The visuospatial function data were extracted from the Rey Complex Figure Test (CFT) and the Clock Drawing Test (CDT). The individual raw score of each neuropsychological test was transformed to normalized Z scores. Subsequently, the normalized Z score was averaged to calculate the composite Z score of each cognitive domain.

### S.3 Operation steps and parameter settings of Spectral dynamic causal modeling

Using SPM (http://www.fil.ion.ucl.ac.uk/spm/software/spm12/), spDCM analysis was conducted. Firstly, specify 1st-level was performed: setting the output directory、time parameters (interscan interval set to 2; Microtime resolution set to 16; Microtime onset set to 8)、the data input consisted of 230 files representing all time points and regression covariates (including white matter, cerebrospinal fluid, and Jenkinson motion parameters). Then, ROI coordinates were inputted and ROIs were defined as spherical regions with a diameter of 6mm in order to extract the time series of each ROI. Subsequently, the full connectivity model space was defined using the previously generated specify 1st-level results and ROI time series. After completing the model construction, Bayesian statistical methods were used to estimate model parameters by evaluating the posterior probability of the model. Finally, the optimal model and its effective connectivity patterns were obtained.

### Supplementary Results

***Supplementary Table 1 (related to Table 1)*.** Detailed raw scores of individual neuropsychological tests for all subjects.

| Items | HC | SCD | aMCI |
| --- | --- | --- | --- |
|  | n=86 | n=72 | n=86 |
| **SCD-Q** | 3.63(1.458) | 6.22(0.915) | 5.17(1.805) |
| **MMSE** | 28.48(1.284) | 28.14(1.447) | 27.03(2.066) |
| **MoCA** | 25.35(2.467) | 25.11(2.053) | 23.03(2.912) |
| **Episodic memory** | | | |
| AVLT-IM | 19.05(4.21) | 18.32(4.24) | 15.21(4.58) |
| AVLT-5-min-DR | 6.22(2.09) | 6.31(2.05) | 4.27(2.43) |
| AVLT-20-min-DR | 6.05(2.11) | 6.30(2.16) | 3.54(2.64) |
| LMT-IR | 6.16(2.99) | 5.36(2.88) | 3.94(2.67) |
| LMT-20-min-DR | 4.89(2.80) | 4.53(2.58) | 2.89(2.26) |
| CFT-20-min-DR | 15.72(6.63) | 16.94(6.45) | 12.19(7.04) |
| **Information processing speed** | | | |
| DSST | 42.00(11.79) | 39.78(11.72) | 33.28(9.02) |
| TMT-A | 55.64(18.16) | 56.78(17.29) | 73.34(37.58) |
| Stoop-A | 27.87(22.16) | 25.74(4.73) | 28.26(5.45) |
| Stoop-B | 41.67(10.81) | 41.64(10.73) | 49.17(17.47) |
| **Executive function** | | | |
| TMT-B | 55.64(18.16) | 74.29(40.53) | 73.36(37.58) |
| Stoop-C | 79.06(23.60) | 77.72(24.99) | 95.06(34.03) |
| DST-backward | 12.63(1.90) | 12.71(2.11) | 11.31(1.92) |
| VFT | 24.13(7.72) | 22.91(4.66) | 19.26(5.13) |
| Semantic Similarity | 19.49(3.65) | 19.24(3.32) | 15.07(4.42) |
| **Visuospatial function** | | | |
| CFT | 34.81(2.02) | 34.64(1.83) | 33.60(3.19) |
| CDT | 9.19(1.47) | 9.24(1.23) | 8.72(1.29) |

Data are presented as the mean (standard deviation, SD). Abbreviations: SCD-Q, Subjective Cognitive Decline Questionnaire; MMSE, Mini-Mental State Exam; MoCA, the Montreal Cognitive Assessment test; AVLT-IM, Auditory Verbal Learning Test-immediate recall; AVLT-5-min-DR, Auditory Verbal Learning Test-5-minute delayed recall; AVLT-20-min-DR, Auditory Verbal Learning Test-20-minute delayed recall; LMT-IR, Logical Memory Test-immediate recall; LMT-20-min-DR, Logical Memory Test-20-minute delayed recall; CFT-20min-DR, Rey-Osterrieth Complex Figure Test-20-minute delayed recall; DSST, Digital Symbol Substitution Test; TMT-A, Trail Making Test-A; Stroop, Stroop Color and Word Test; TMT-B, Trail Making Test-B; DST, Digit Span Test; VFT, Verbal Fluency Test; Similarity, Semantic Similarity Test; CFT, Rey-Osterrieth Complex Figure Test; CDT, Clock Drawing Test.

***Supplementary Table 2 (related to Table 1).*** The p-values of demographics and clinical measures across different groups.

|  | **HC vs. SCD** | **HC vs. aMCI** | **SCD vs. aMCI** |
| --- | --- | --- | --- |
| Age (years) | 0.310 | 0.281 | 1.000 |
| Education level (years) | 0.477 | 0.006^**^ | 0.353 |
| SCD-Q | 0.000^***^ | 0.000^***^ | 0.000^***^ |
| MMSE | 0.511 | 0.000^***^ | 0.001^***^ |
| MoCA | 1.000 | 0.000^***^ | 0.000^***^ |
| Episodic memory | 1.000 | 0.000^***^ | 0.000^***^ |
| Information processing speed | 1.000 | 0.000^***^ | 0.000^***^ |
| Executive function | 1.000 | 0.000^***^ | 0.000^***^ |
| Visuospatial function | 1.000 | 0.001^***^ | 0.004^**^ |

HC, healthy controls; SCD, subjective cognitive decline; aMCI, amnestic mild cognitive impairment; SCD-Q, Subjective Cognitive Decline Questionnaire; MMSE, Mini-Mental State Exam; MoCA, the Montreal Cognitive Assessment test; Bonferroni correction was applied for multiple group comparisons. *p ≤ 0.05, ** p ≤ 0.01, ***p ≤ 0.001.

***Supplementary Table 3 (related to Table 2).*** Detailed raw scores of individual neuropsychological tests for all subjects.

| Items | Before rTMS SCD | After rTMS SCD | Before rTMS aMCI | After rTMS aMCI |
| --- | --- | --- | --- | --- |
|  | n=10 | n=10 | n=11 | n=11 |
| **MMSE** | 27.9(2.644) | 28.30(1.767) | 27.09(1.700) | 27.91(1.044) |
| **Episodic memory** | | | | |
| AVLT-IM | 17.50(3.89) | 24.70(7.73) | 14.64(3.93) | 21.91(5.47) |
| AVLT-5-min-DR | 6.40(2.49) | 8.80(3.11) | 4.91(2.39) | 7.55(2.42) |
| AVLT-20-min-DR | 6.40(2.99) | 7.90(3.81) | 4.27(3.23) | 7.45(2.62) |
| LMT-IR | 5.20(2.82) | 6.90(3.21) | 5.64(3.78) | 7.09(2.95) |
| LMT-20-min-DR | 4.00(2.91) | 5.80(2.44) | 4.73(3.69) | 5.45(2.50) |
| CFT-20-min-DR | 18.30(7.45) | 24.20(8.66) | 10.55(7.74) | 16.91(8.56) |
| **Information processing speed** | | | | |
| DSST | 37.60(8.67) | 41.30(8.24) | 32.09(9.43) | 36.45(10.85) |
| TMT-A | 49.60(10.02) | 55.44(21.04) | 79.36(26.08) | 64.45(21.11) |
| Stoop-A | 26.50(5.34) | 26.00(6.42) | 28.27(8.75) | 28.18(5.31) |
| Stoop-B | 44.40(17.37) | 38.22(8.45) | 45.82(12.55) | 42.45(11.00) |
| **Executive function** | | | | |
| TMT-B | 163.20(63.06) | 129.22(48.74) | 190.00(84.75) | 181.55(75.15) |
| Stoop-C | 78.50(22.81) | 69.00(20.45) | 92.18(27.08) | 78.55(27.34) |
| DST-backward | 13.10(1.52) | 11.90(1.79) | 10.64(0.92) | 11.00(1.61) |
| VFT | 21.10(4.50) | 23.30(6.79) | 20.27(6.06) | 23.32(4.85) |
| Semantic Similarity | 19.30(3.97) | 20.30(3.53) | 16.27(4.69) | 19.64(5.18) |
| **Visuospatial function** | | | | |
| CFT | 34.7(2.00) | 34.60(2.84) | 30.73(4.52) | 33.18(3.46) |
| CDT | 9.60(0.84) | 9.20(1.03) | 9.27(0.79) | 9.36(0.50) |

Data are presented as the mean (standard deviation, SD). Abbreviations: MMSE, Mini-Mental State Exam; AVLT-IM, Auditory Verbal Learning Test-immediate recall; AVLT-5-min-DR, Auditory Verbal Learning Test-5-minute delayed recall; AVLT-20-min-DR, Auditory Verbal Learning Test-20-minute delayed recall; LMT-IR, Logical Memory Test-immediate recall; LMT-20-min-DR, Logical Memory Test-20-minute delayed recall; CFT-20min-DR, Rey-Osterrieth Complex Figure Test-20-minute delayed recall; DSST, Digital Symbol Substitution Test; TMT-A, Trail Making Test-A; Stroop, Stroop Color and Word Test; TMT-B, Trail Making Test-B; DST, Digit Span Test; VFT, Verbal Fluency Test; Similarity, Semantic Similarity Test; CFT, Rey-Osterrieth Complex Figure Test; CDT, Clock Drawing Test.

***Supplementary Figure.*** 27 independent component images.


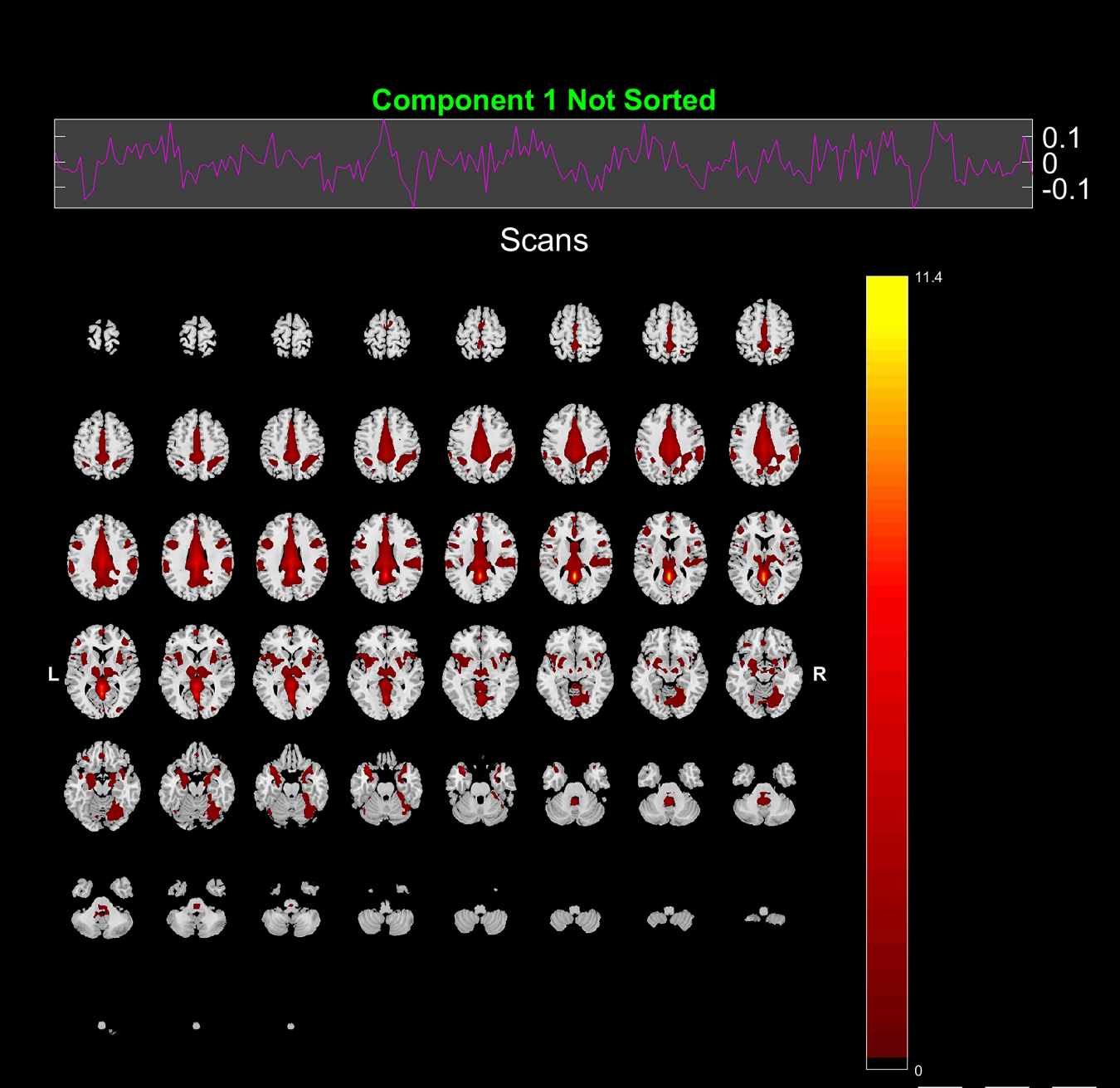


correlations with the DMN templates: r = 0.41194

correlations with the CEN templates: r = 0.040927


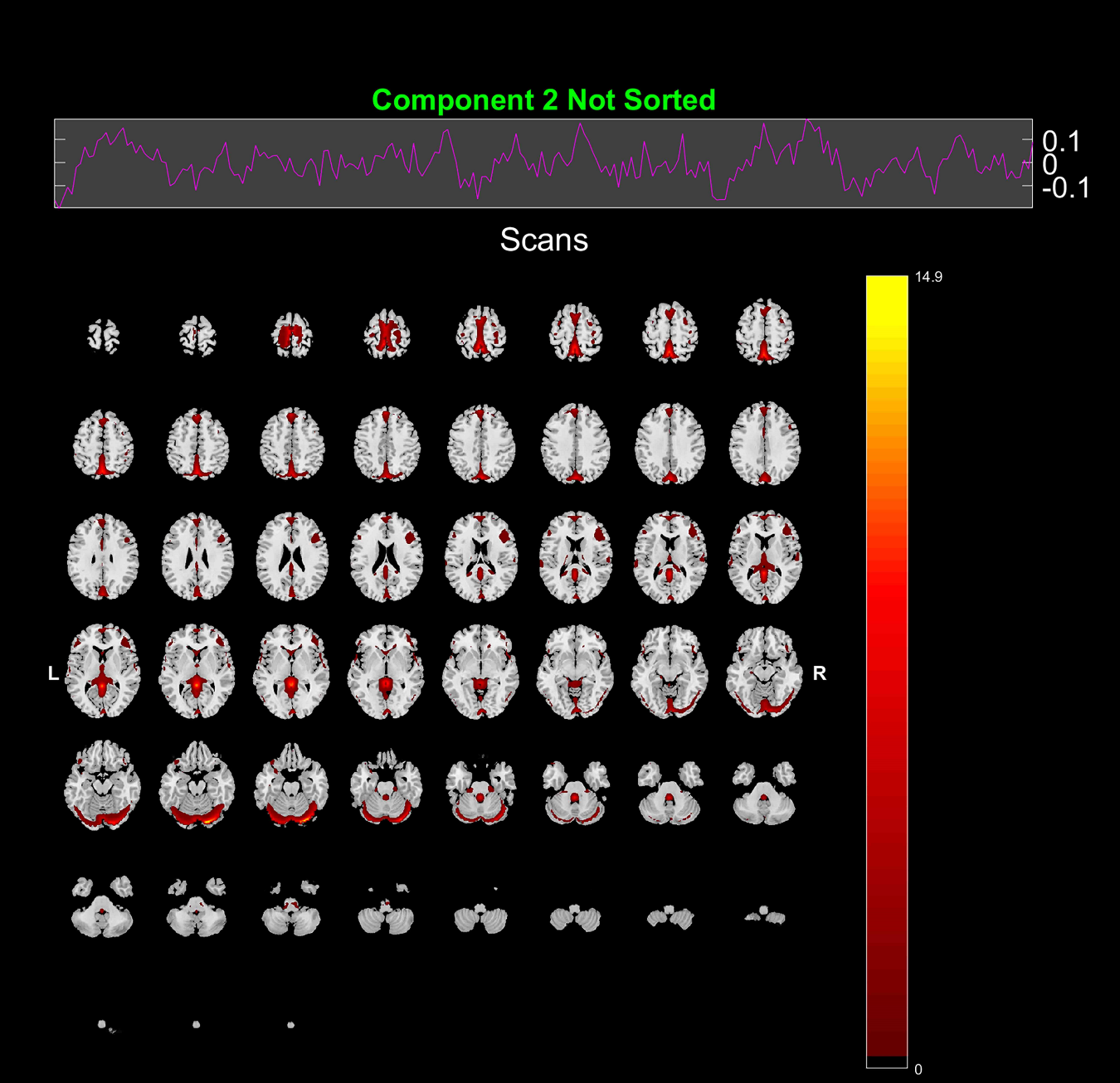


correlations with the DMN templates: r = 0.17348

correlations with the CEN templates: r = 0.0073663


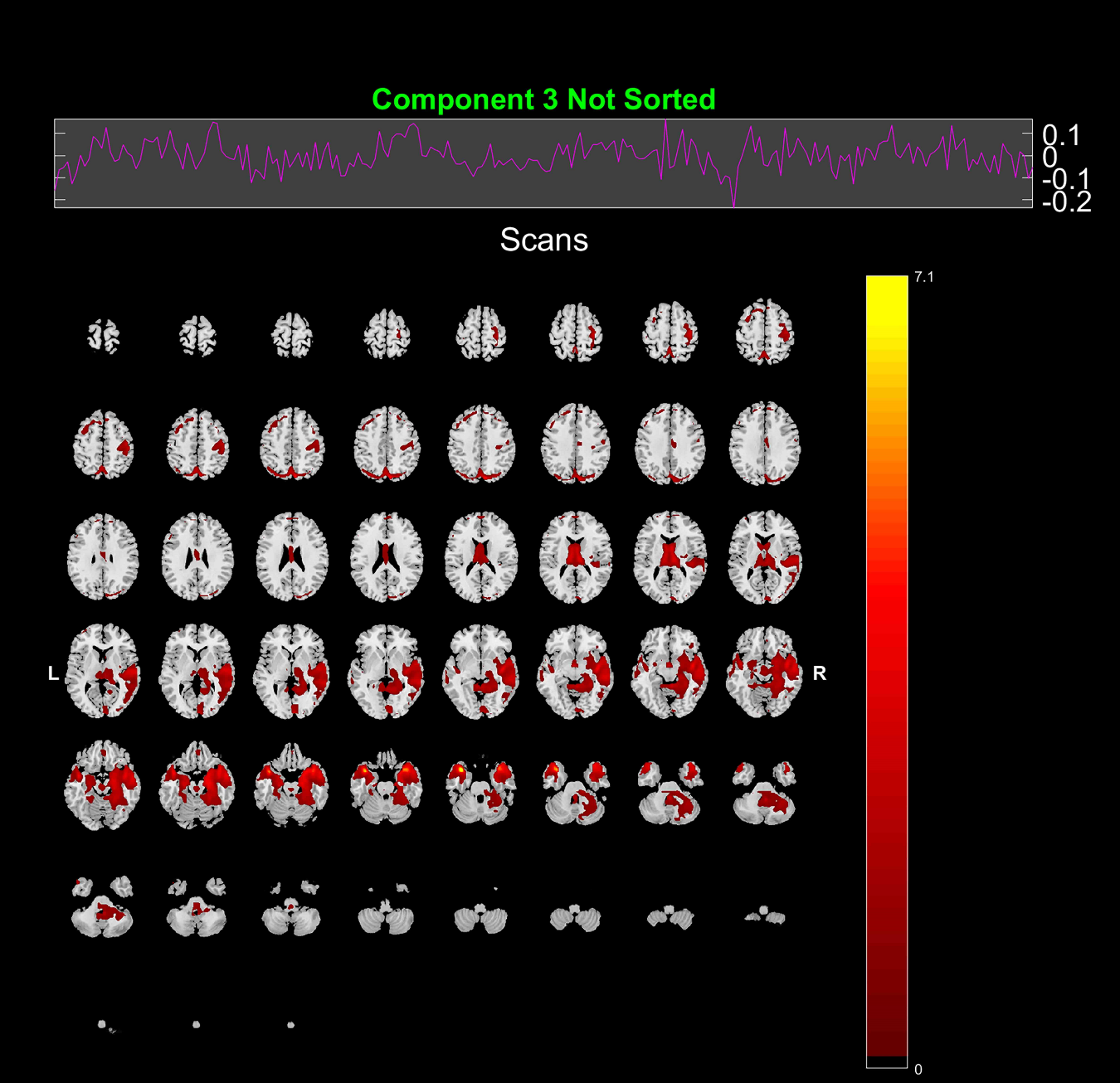


correlations with the DMN templates: r = 0.039567

correlations with the CEN templates: r = 0.0036974


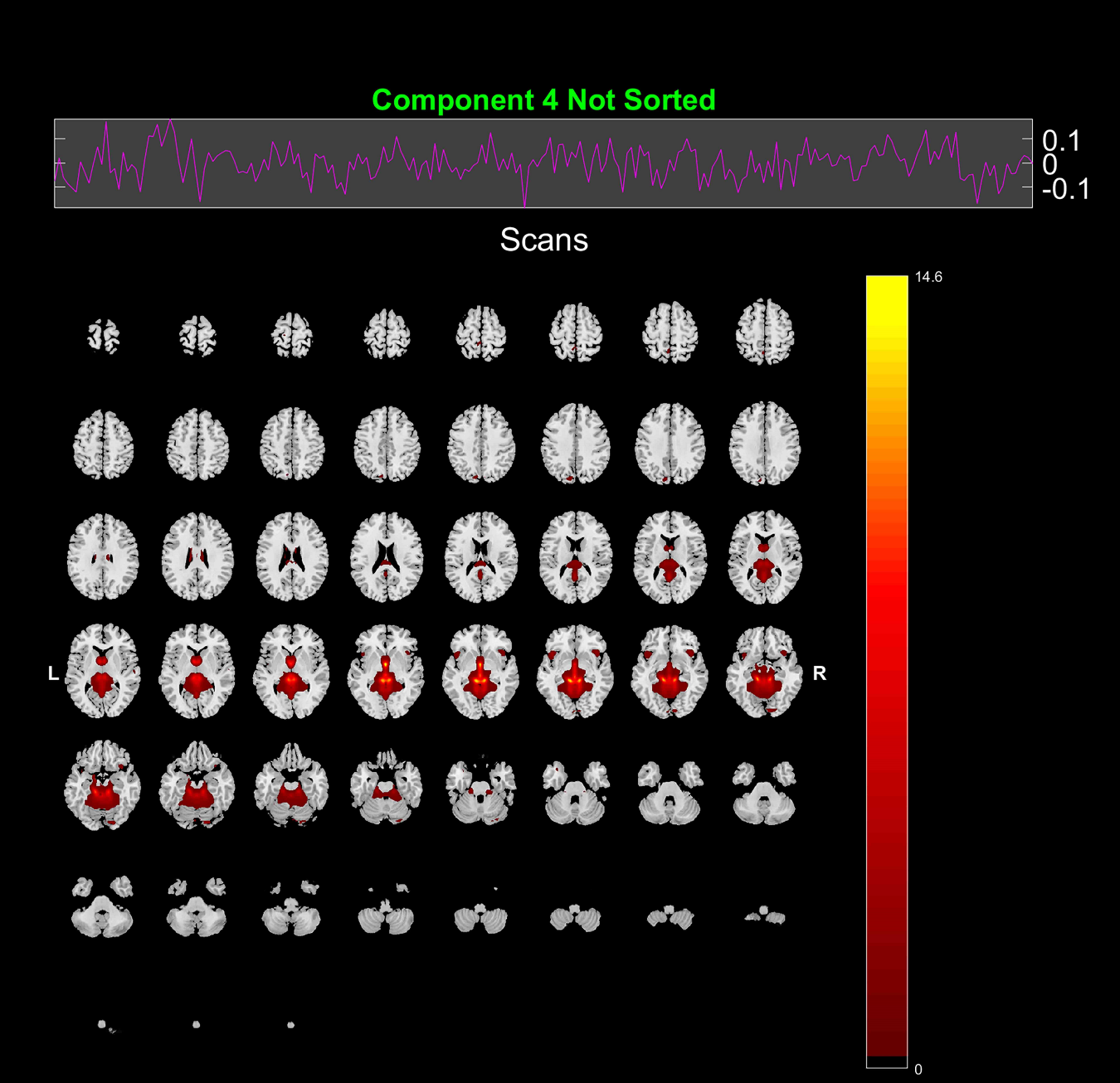


correlations with the DMN templates: r = 0.072643

correlations with the CEN templates: r = 0.01782


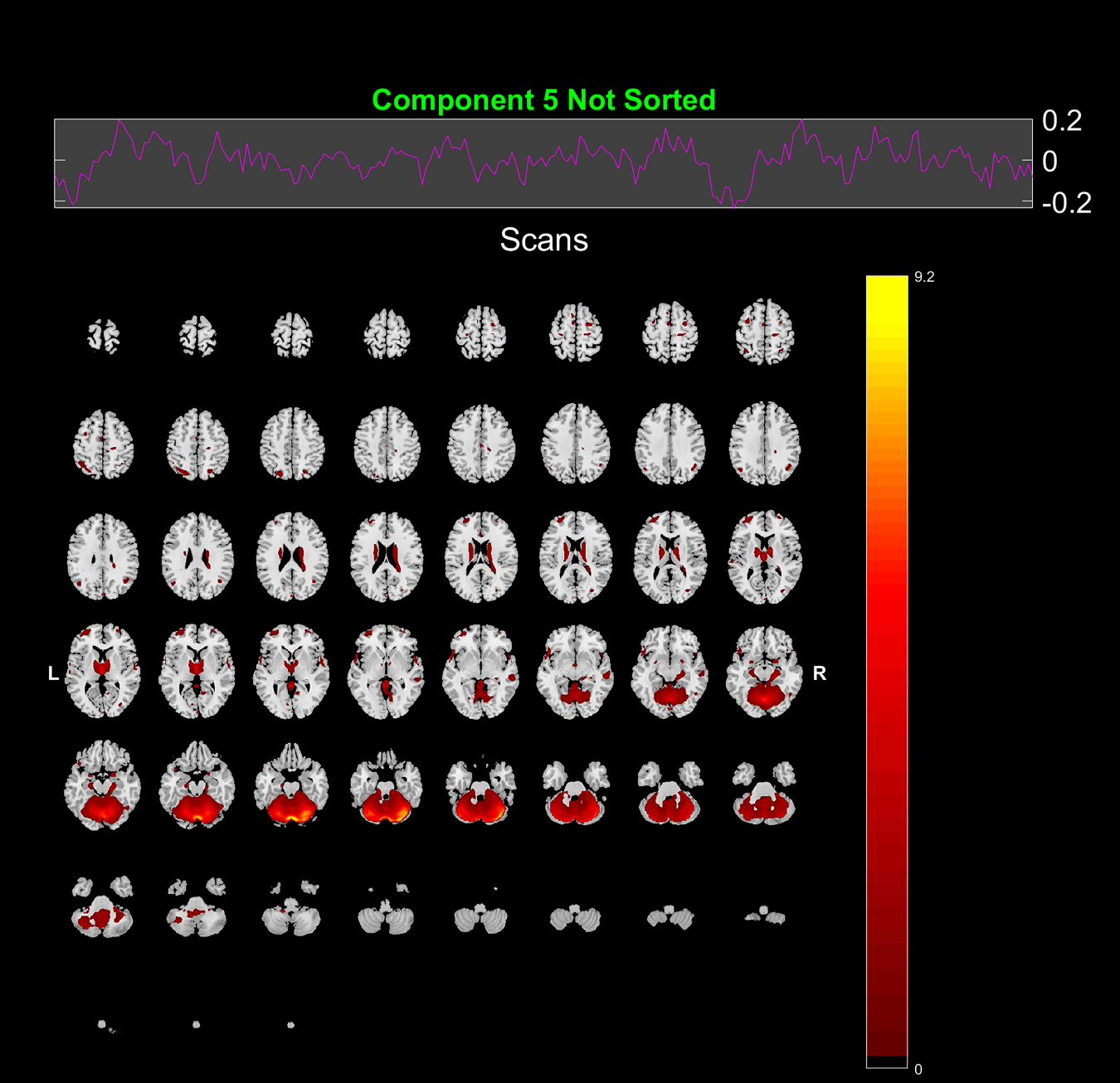


correlations with the DMN templates: r = 0.041089

correlations with the CEN templates: r = 0.038858


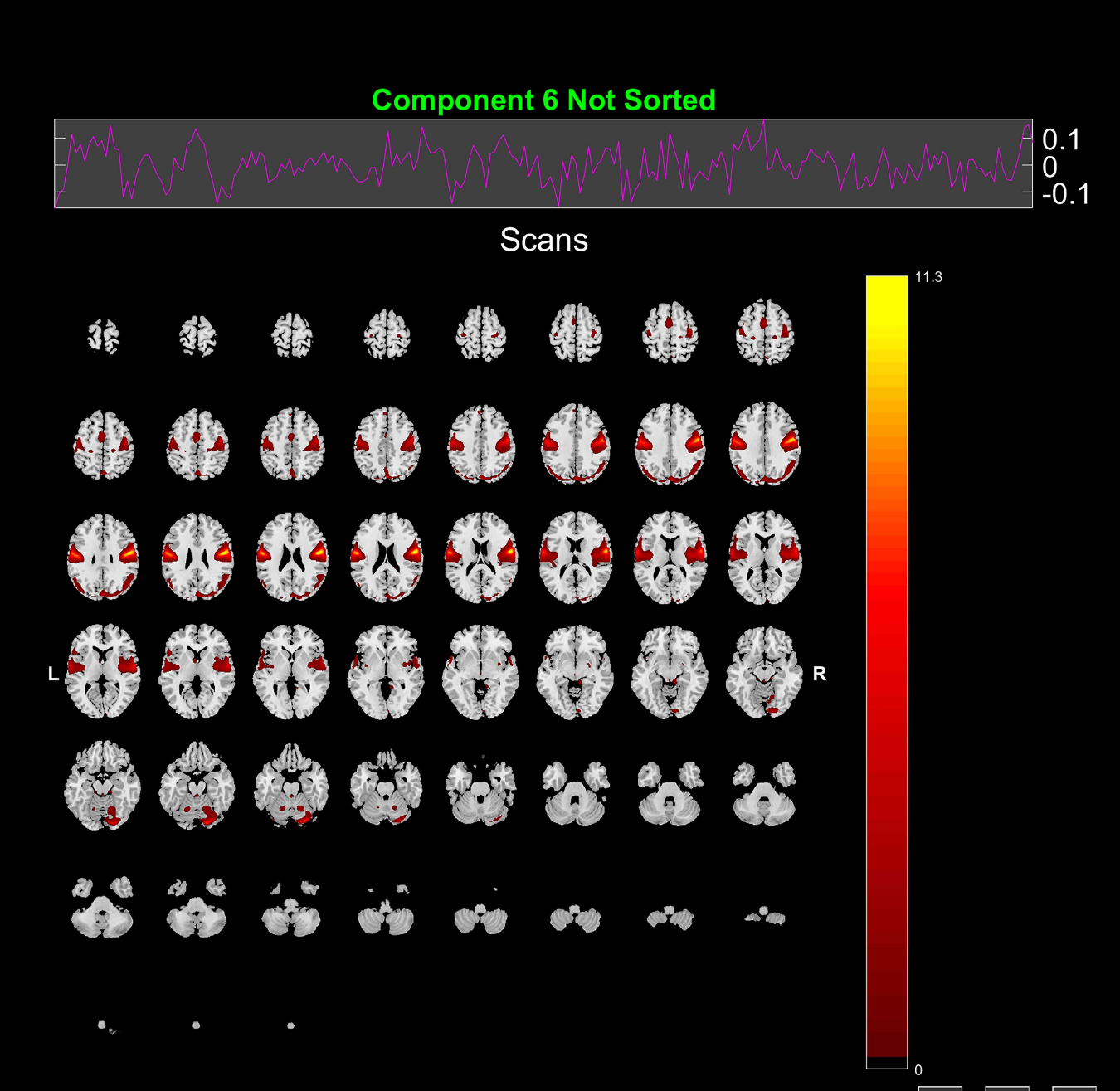


correlations with the DMN templates: r = 0.052498

correlations with the CEN templates: r = 0.045152


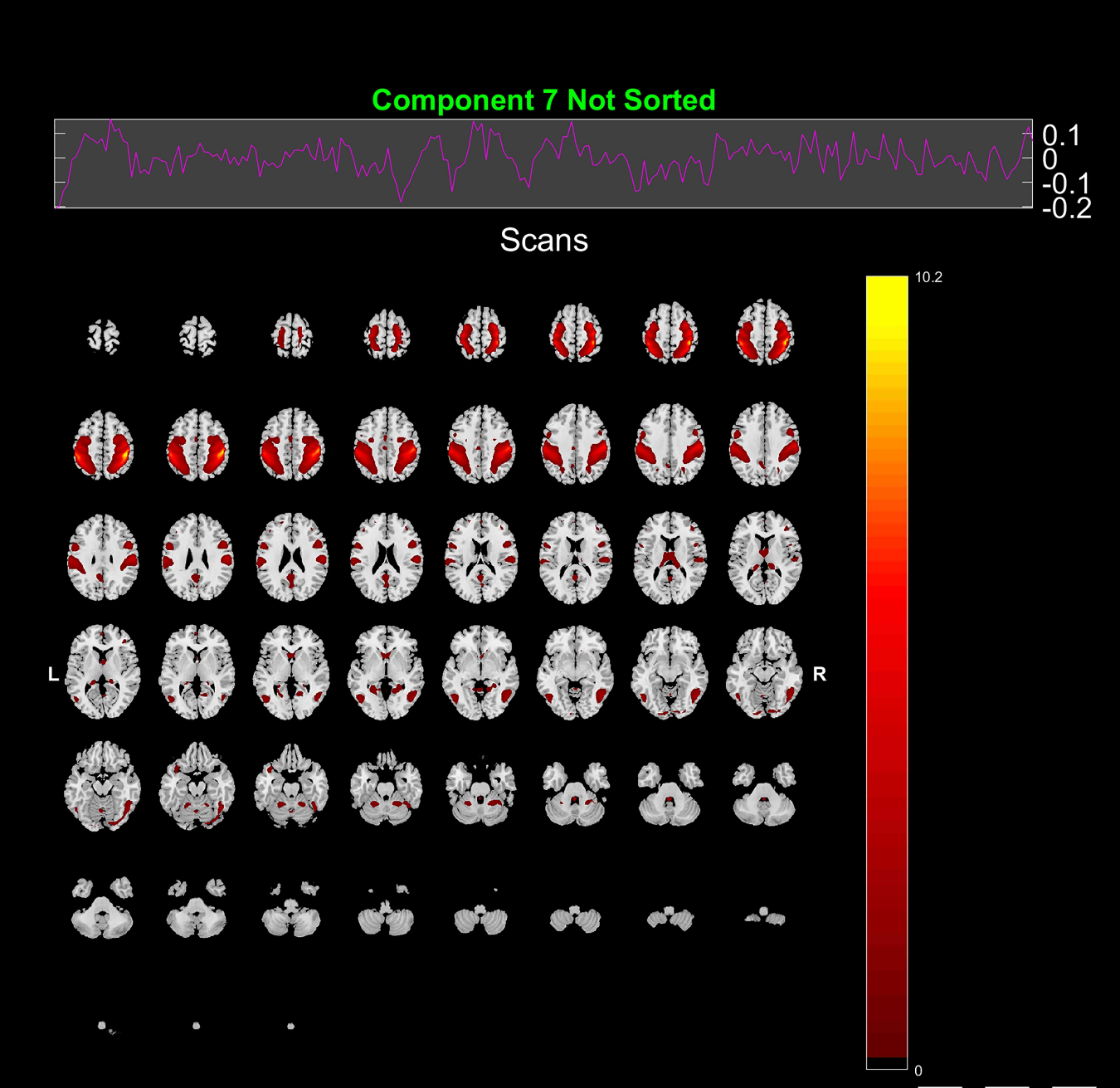


correlations with the DMN templates: r = 0.029515

correlations with the CEN templates: r = 0.15439


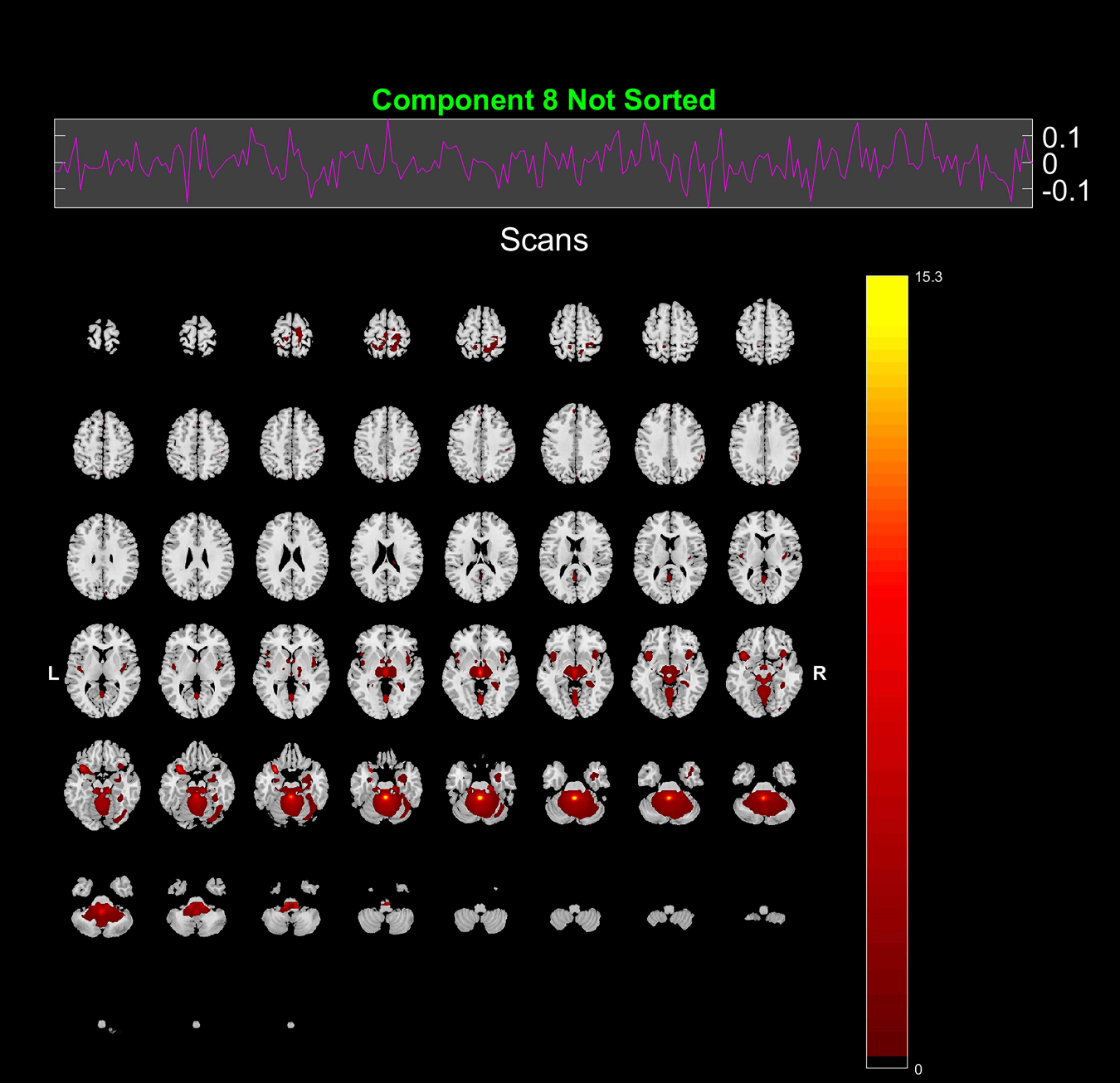


correlations with the DMN templates: r = 0.014556

correlations with the CEN templates: r = 0.0066


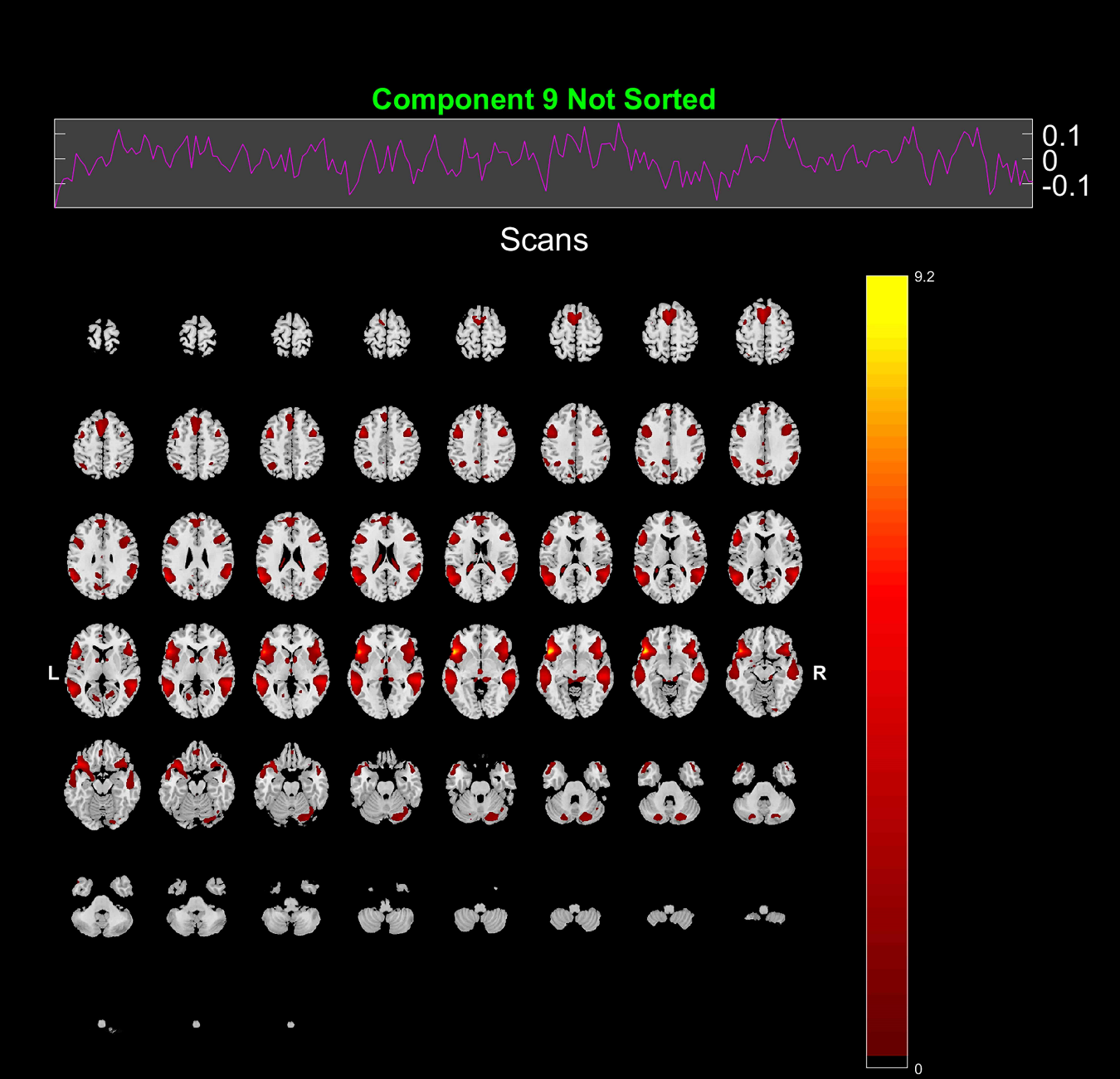


correlations with the DMN templates: r = 0.14729

correlations with the CEN templates: r = 0.27236


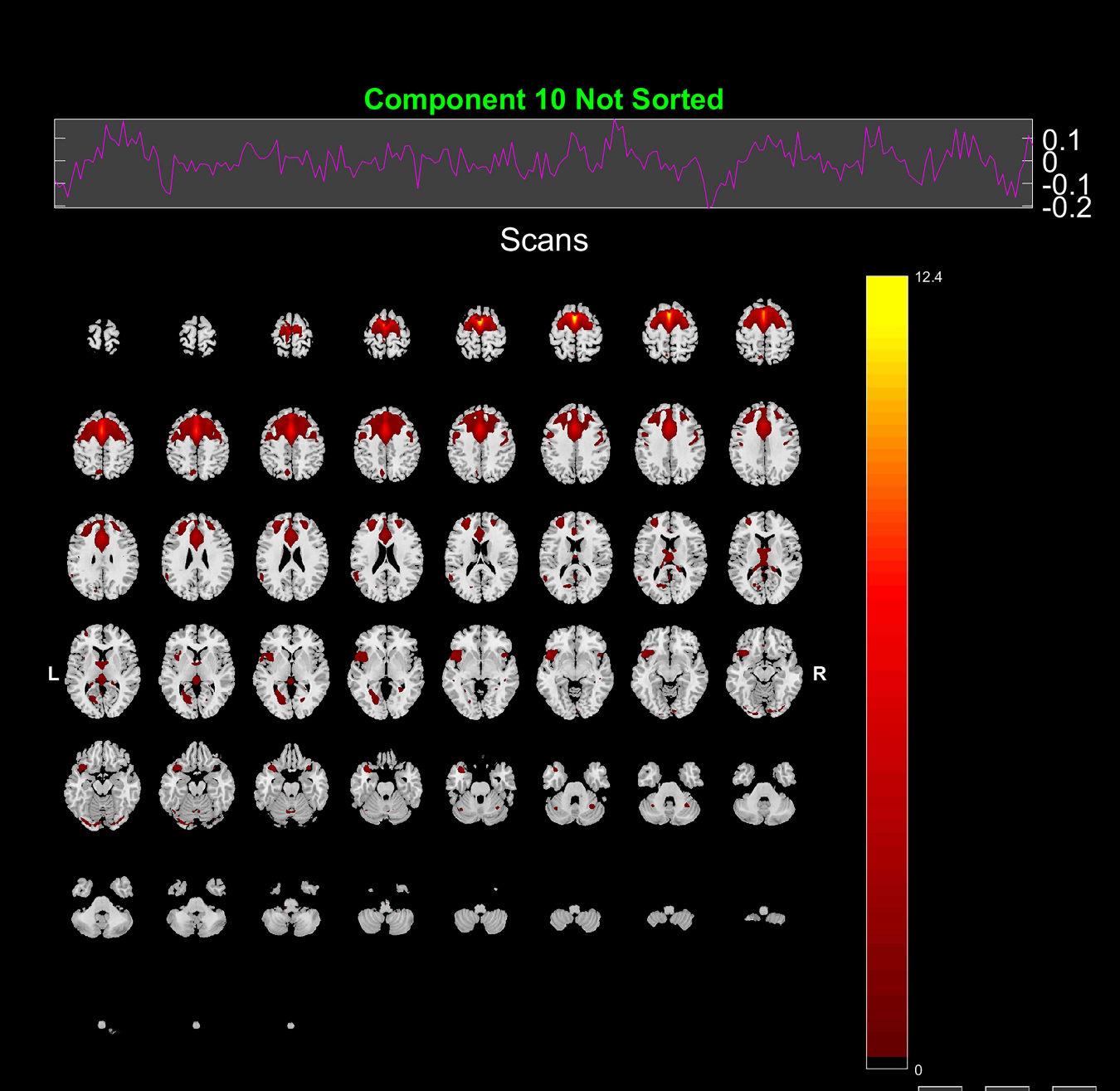


correlations with the DMN templates: r = 0.021278

correlations with the CEN templates: r = 0.15274


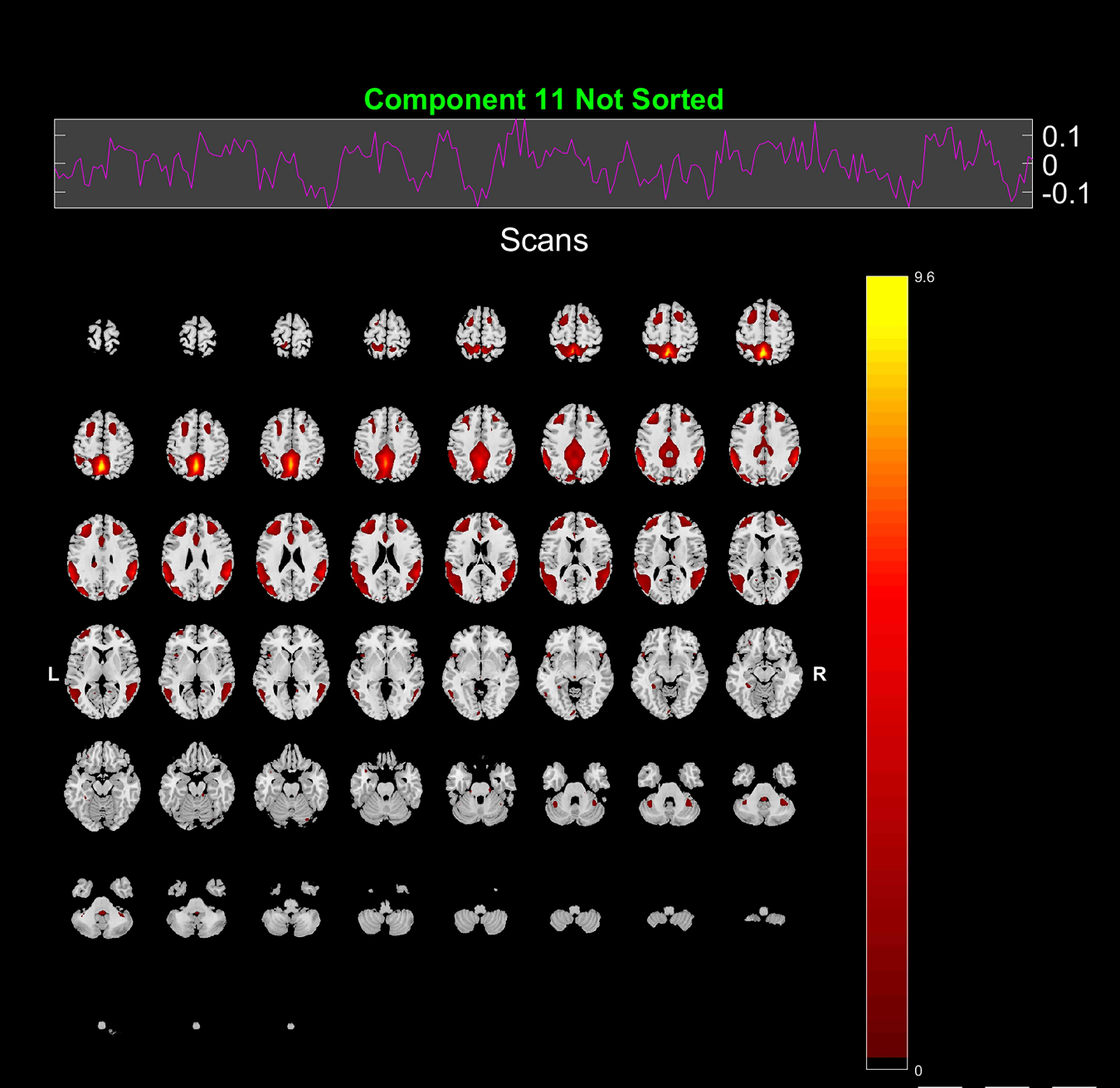
correlations with the DMN templates: r = 0.35233

correlations with the CEN templates: r = 0.089898


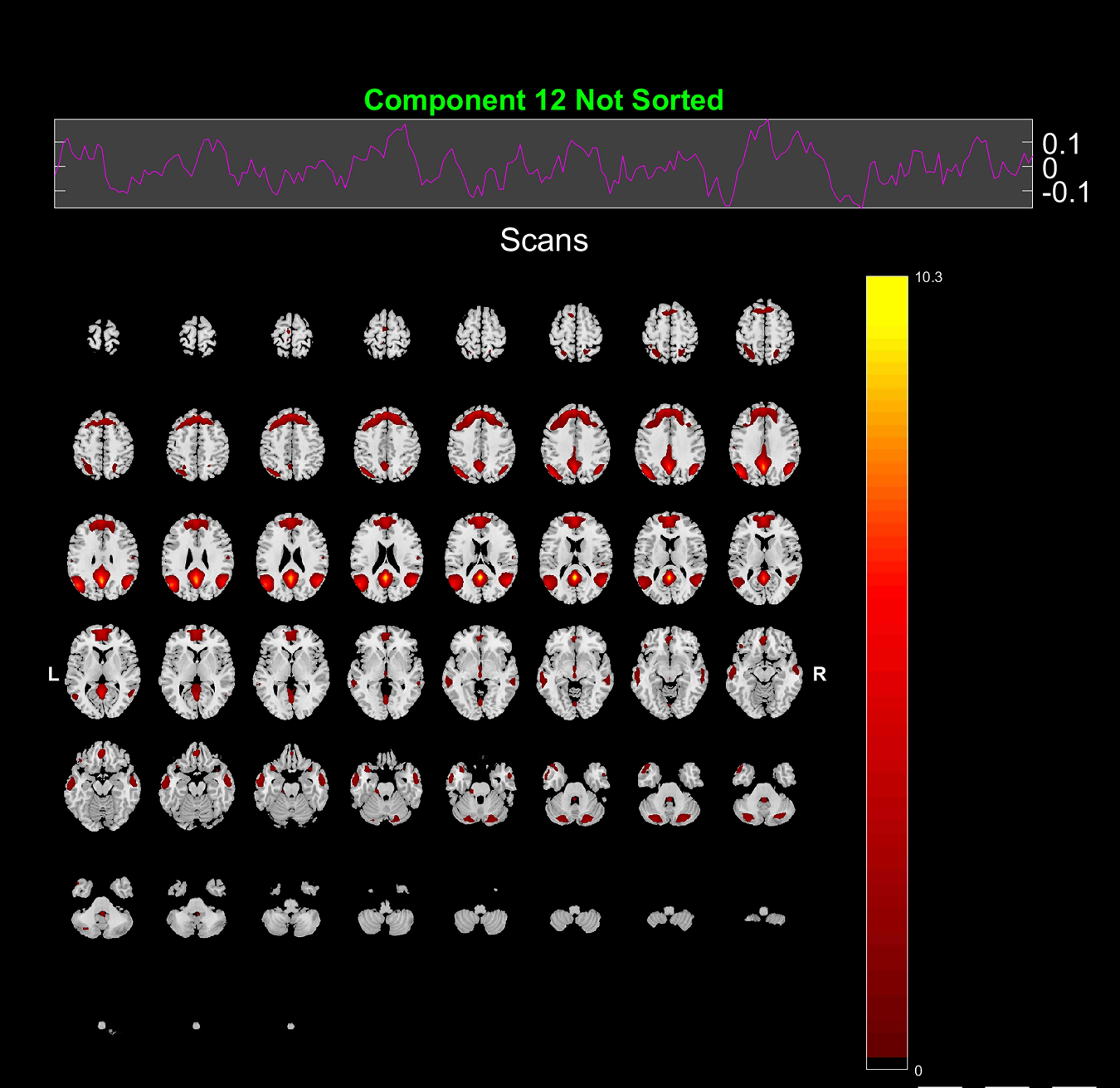


correlations with the DMN templates: r = 0.39147

correlations with the CEN templates: r = 0.013268


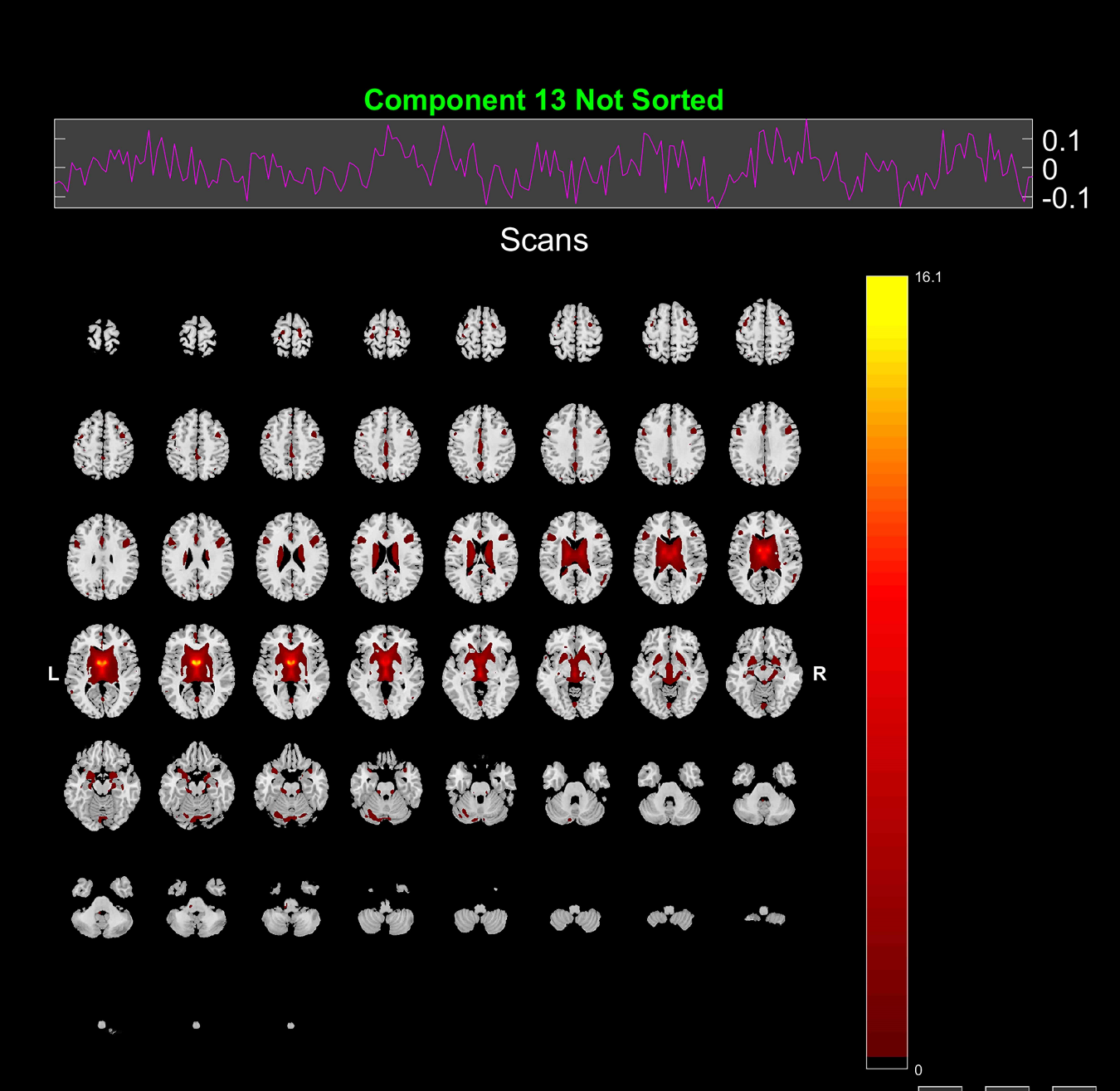


correlations with the DMN templates: r = 0.042315

correlations with the CEN templates: r = 0.0020509


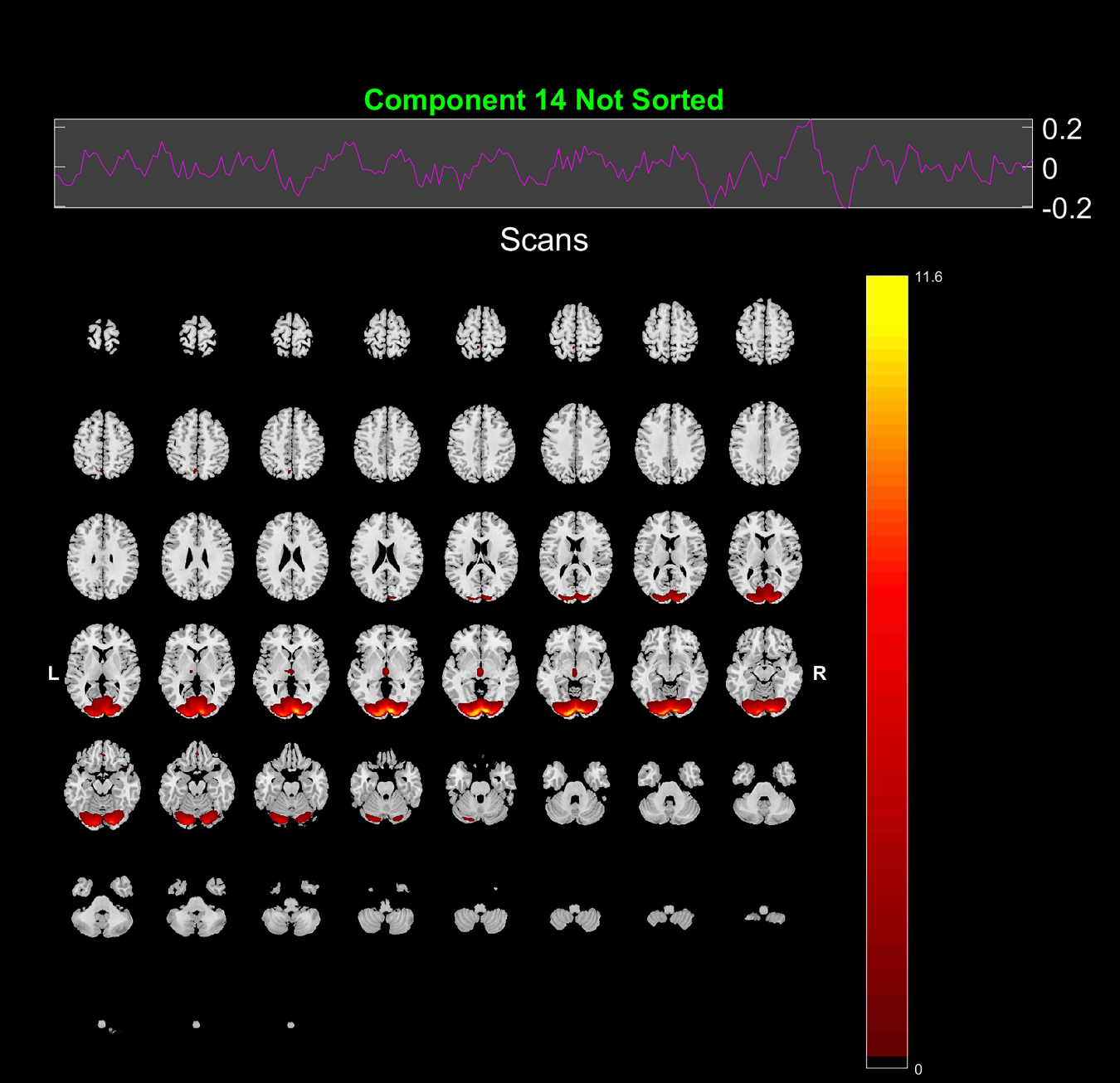


correlations with the DMN templates: r = 0.053572

correlations with the CEN templates: r = 0.023558


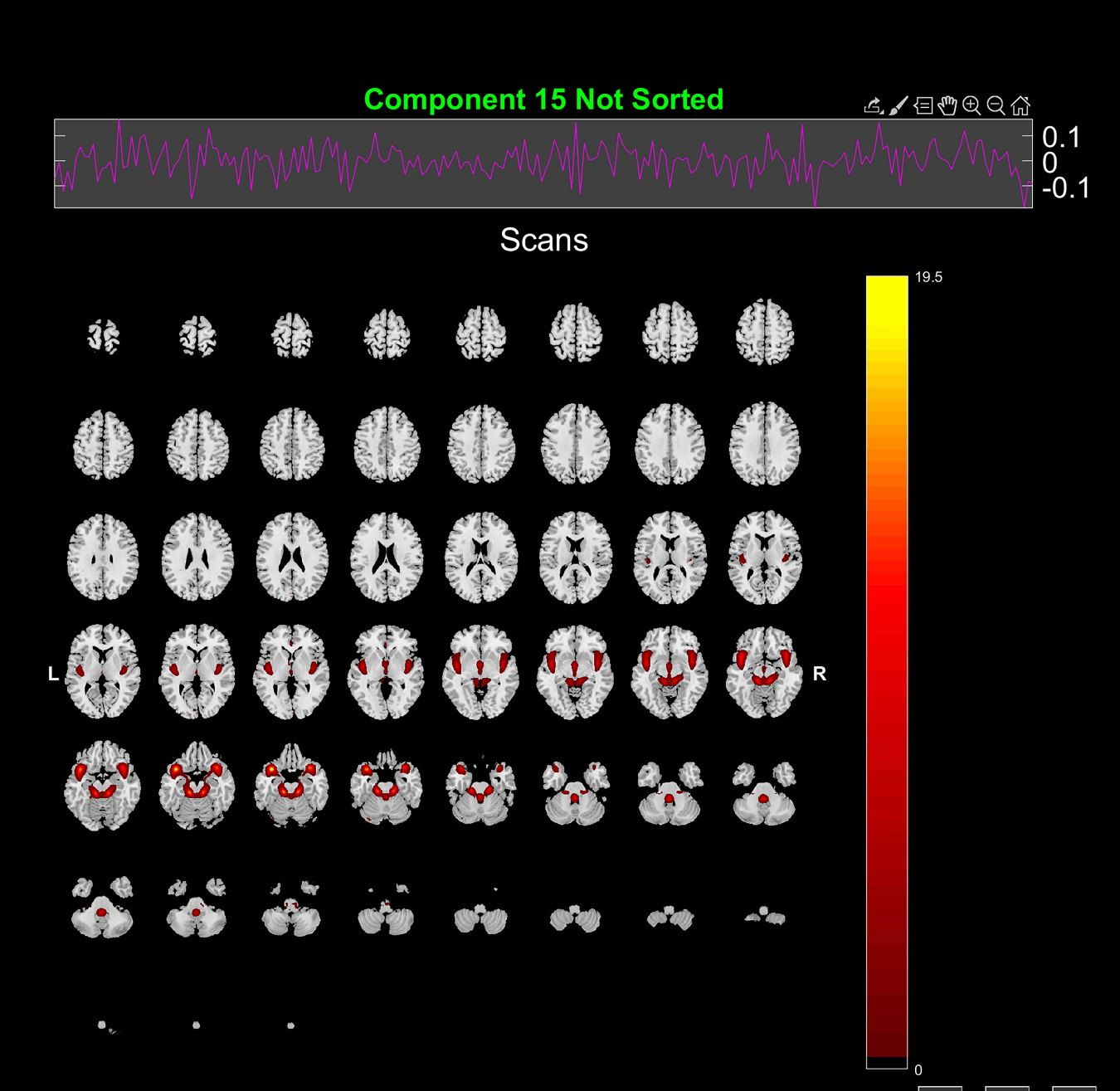


correlations with the DMN templates: r = -0.0041455

correlations with the CEN templates: r = 0.003575


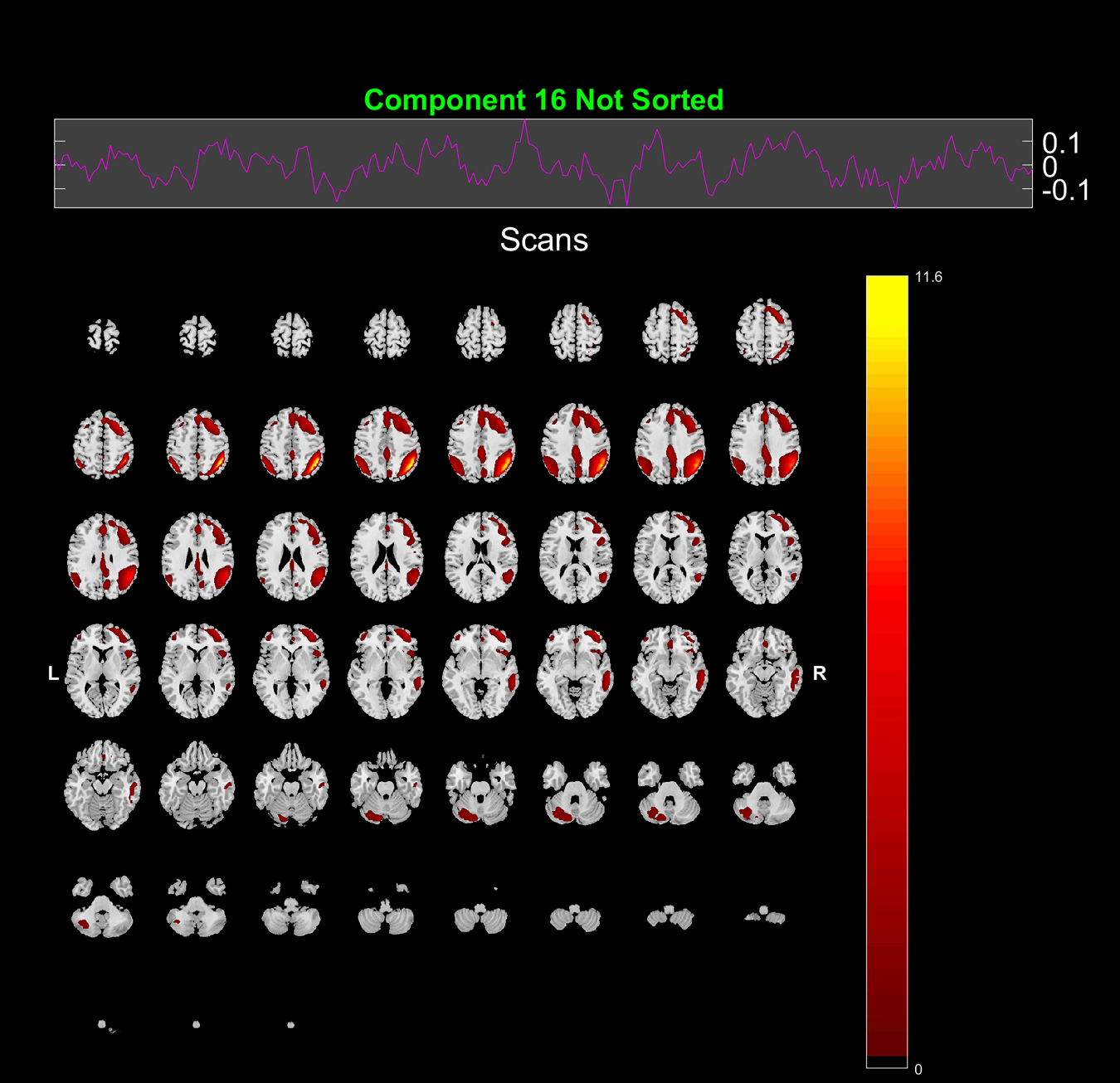


correlations with the DMN templates: r = 0.22938

correlations with the CEN templates: r = 0.37048


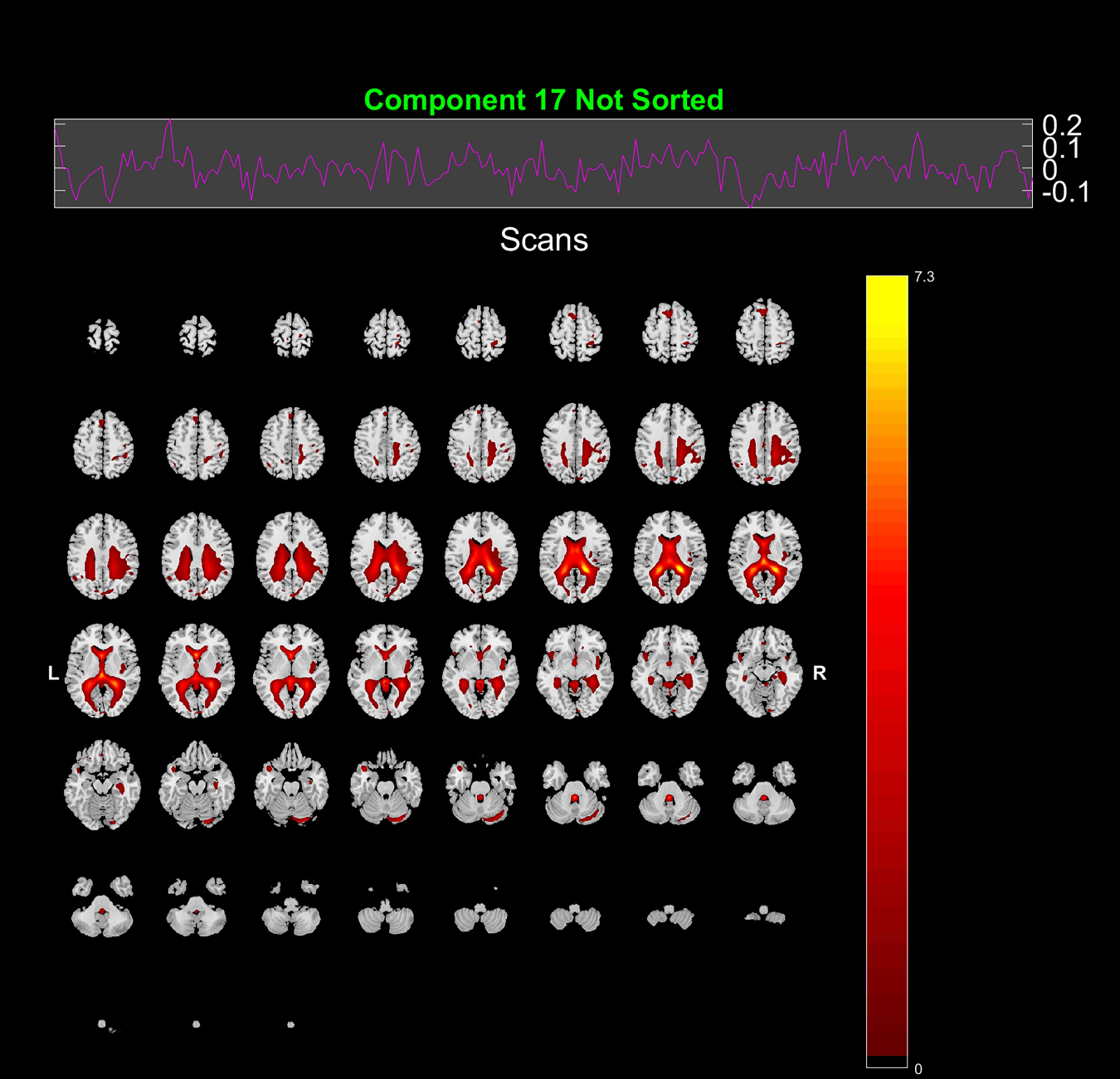


correlations with the DMN templates: r = 0.069433

correlations with the CEN templates: r = 0.058521


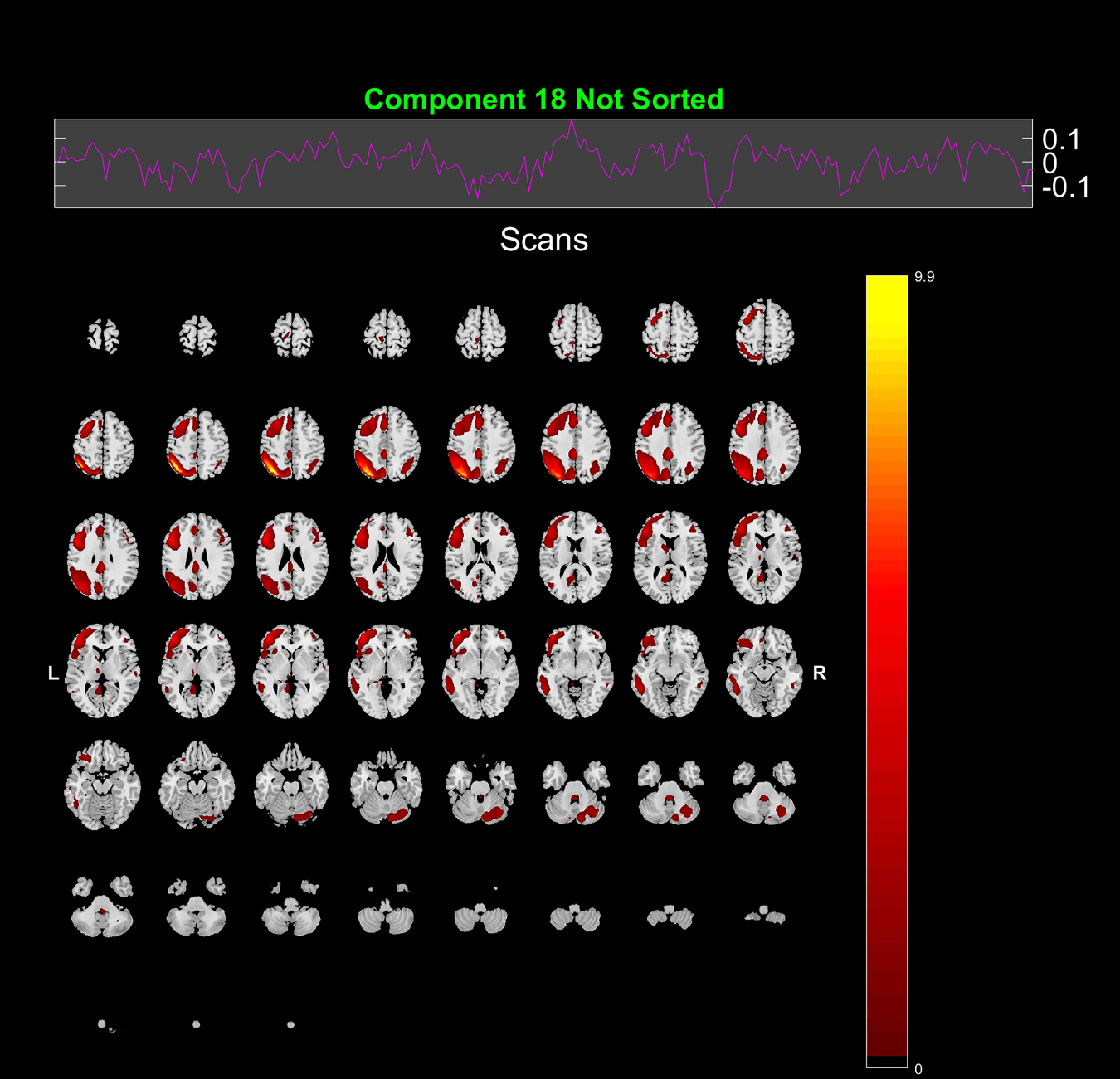


correlations with the DMN templates: r = 0.12776

correlations with the CEN templates: r = 0.37276


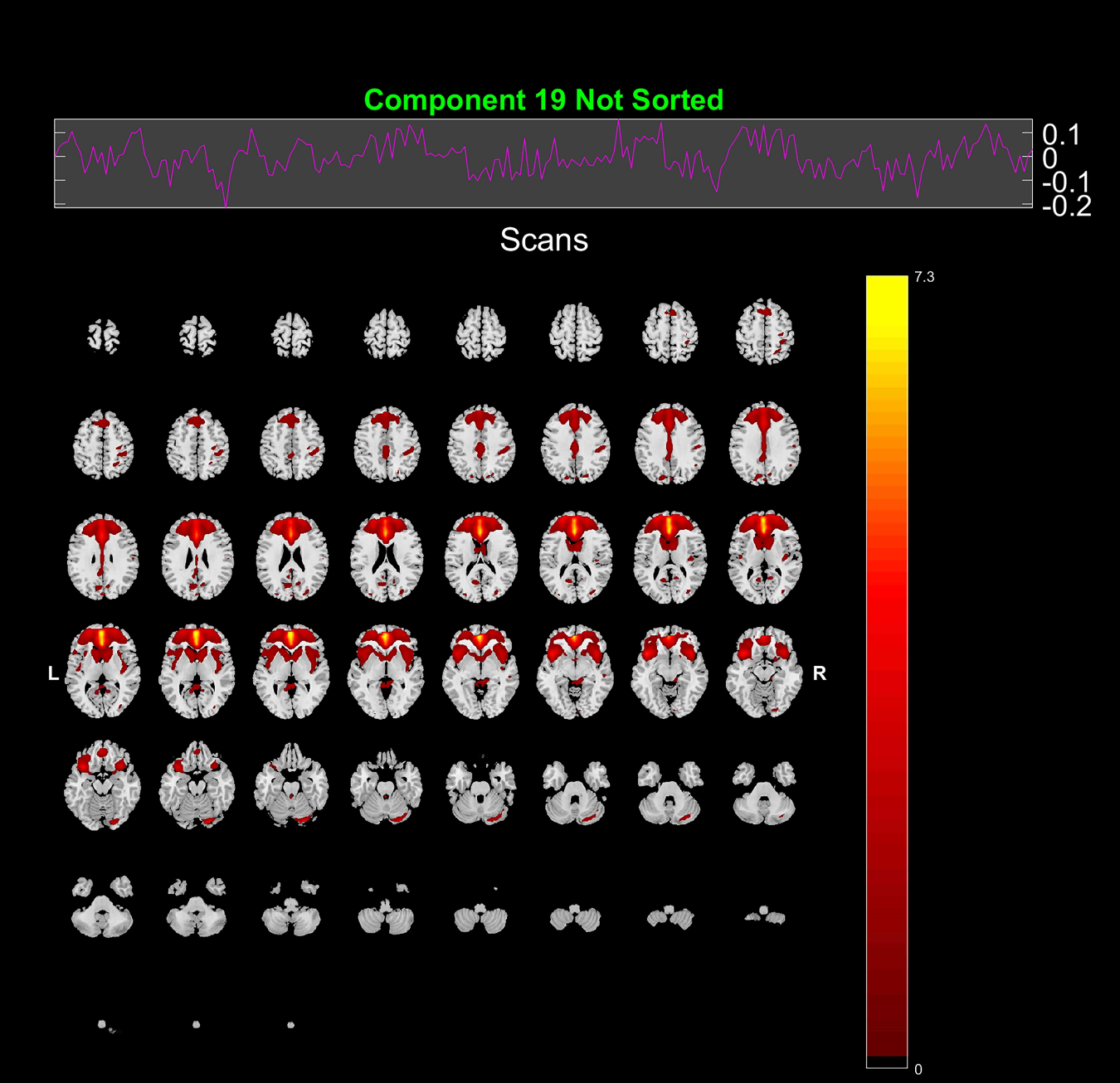


correlations with the DMN templates: r = 0.070636

correlations with the CEN templates: r = 0.094818


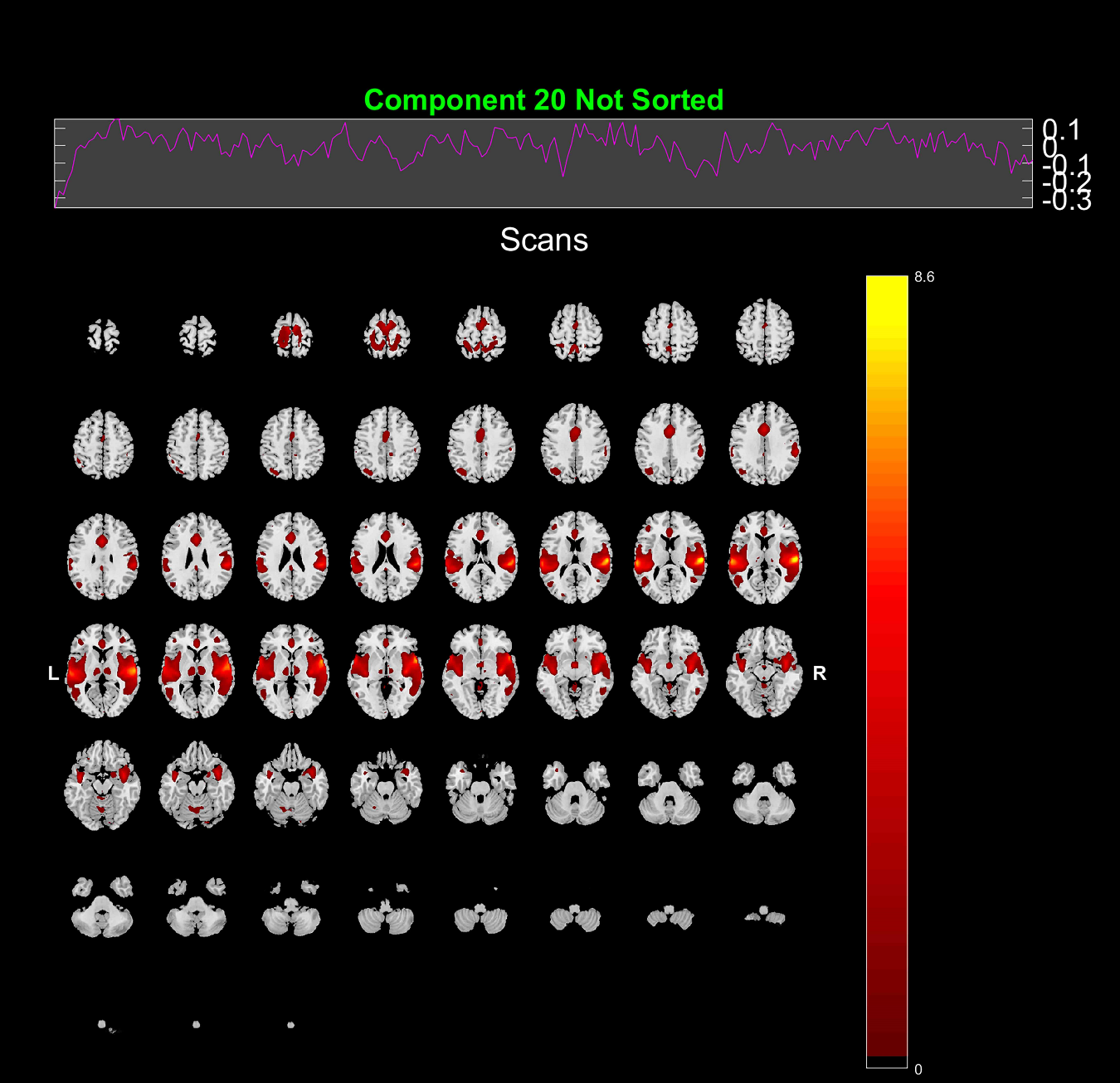


correlations with the DMN templates: r = 0.015672

correlations with the CEN templates: r = 0.00081644


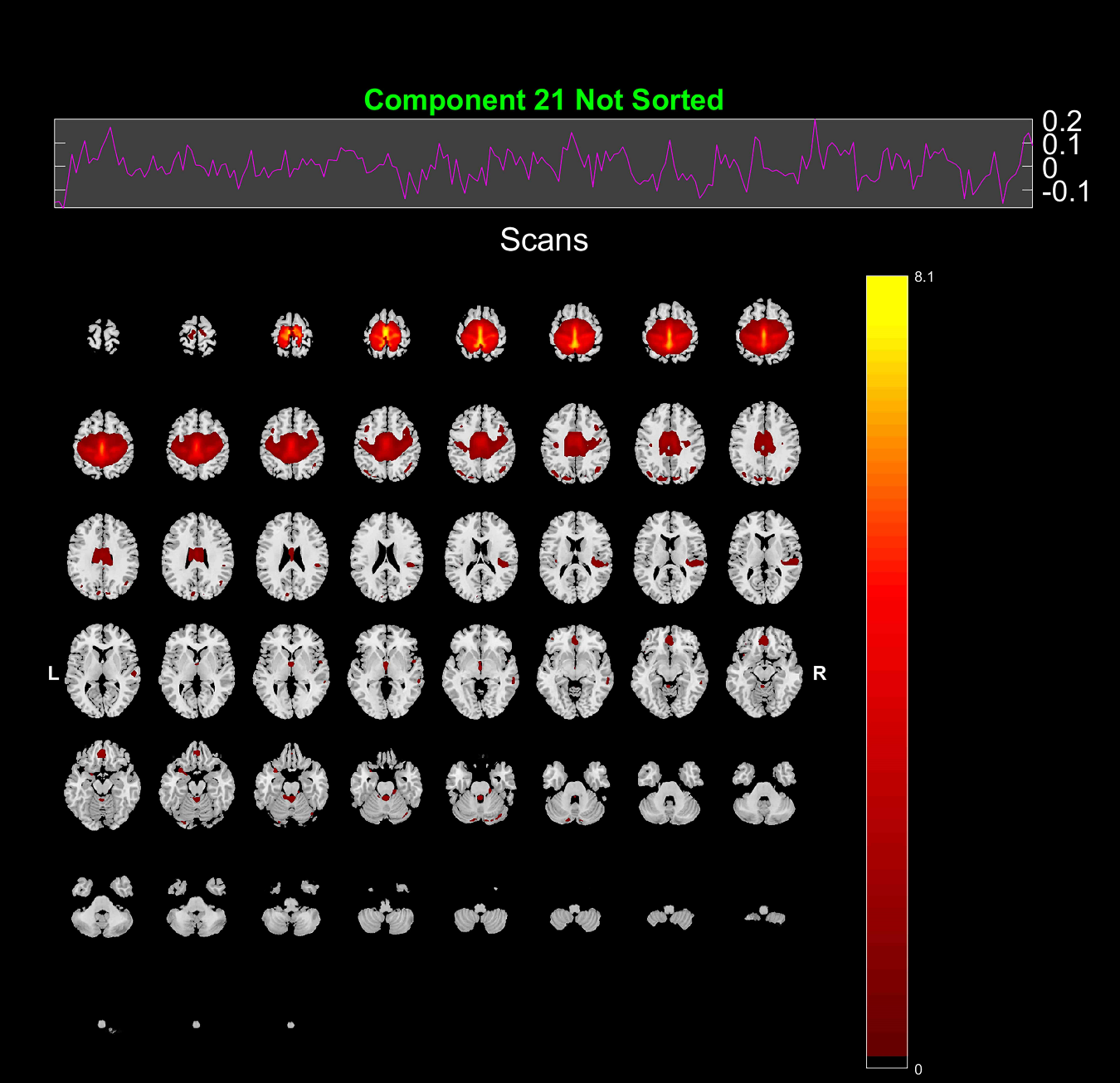


correlations with the DMN templates: r = 0.23158

correlations with the CEN templates: r = 0.022786


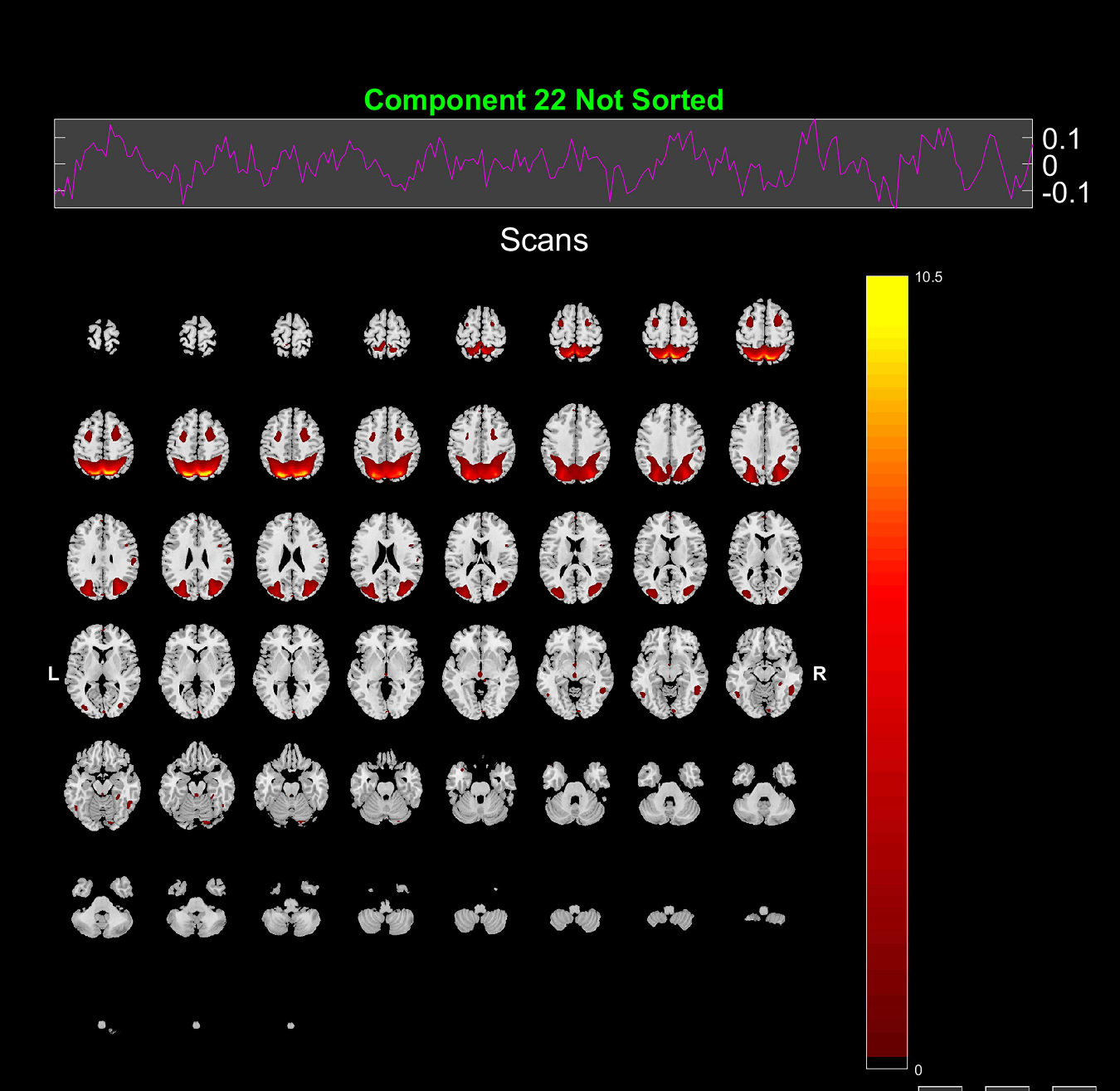


correlations with the DMN templates: r = 0.10139

correlations with the CEN templates: r = 0.15475


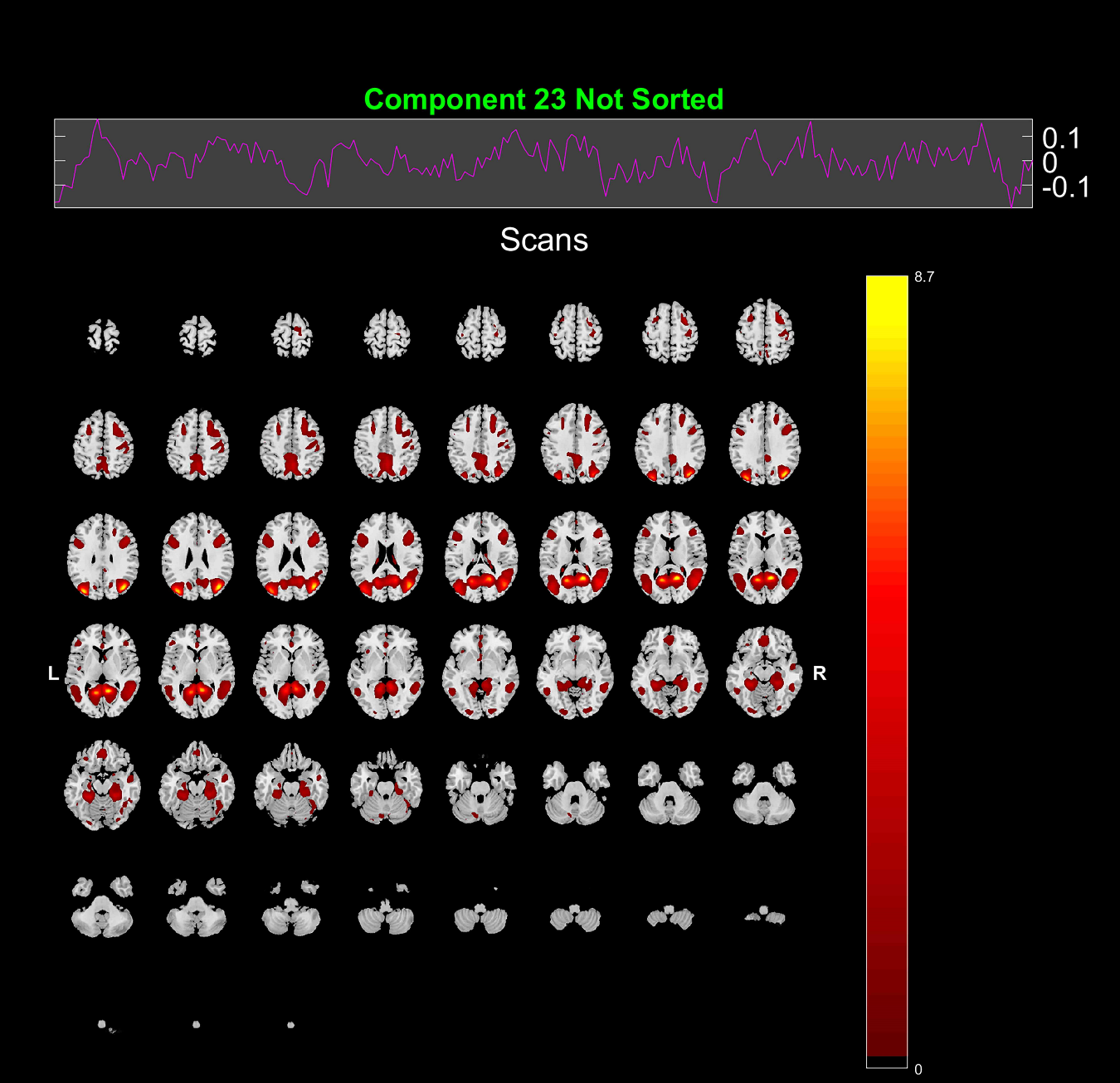


correlations with the DMN templates: r = 0.20584

correlations with the CEN templates: r = 0.11586


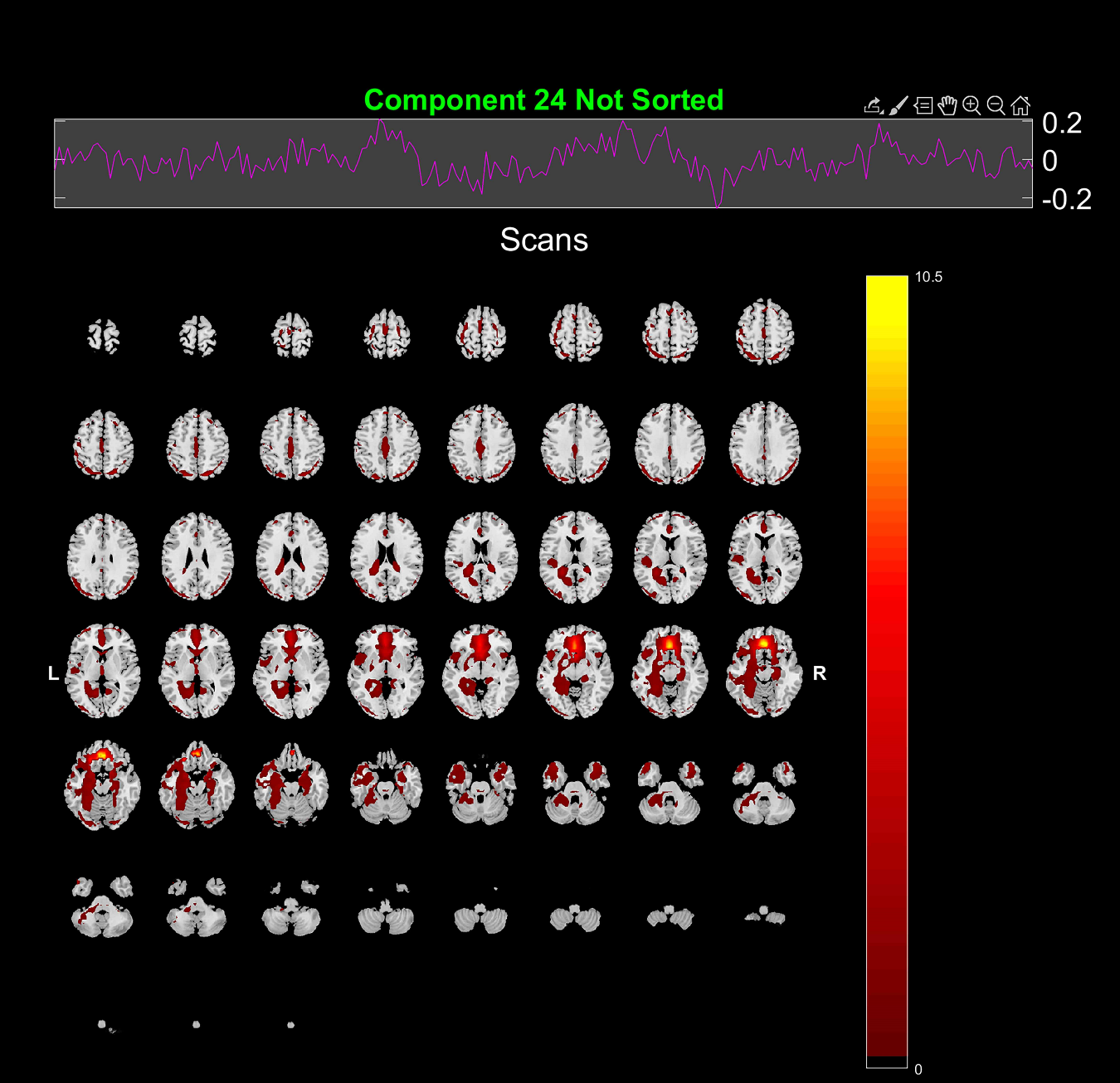


correlations with the DMN templates: r = 0.10221

correlations with the CEN templates: r = 0.014932


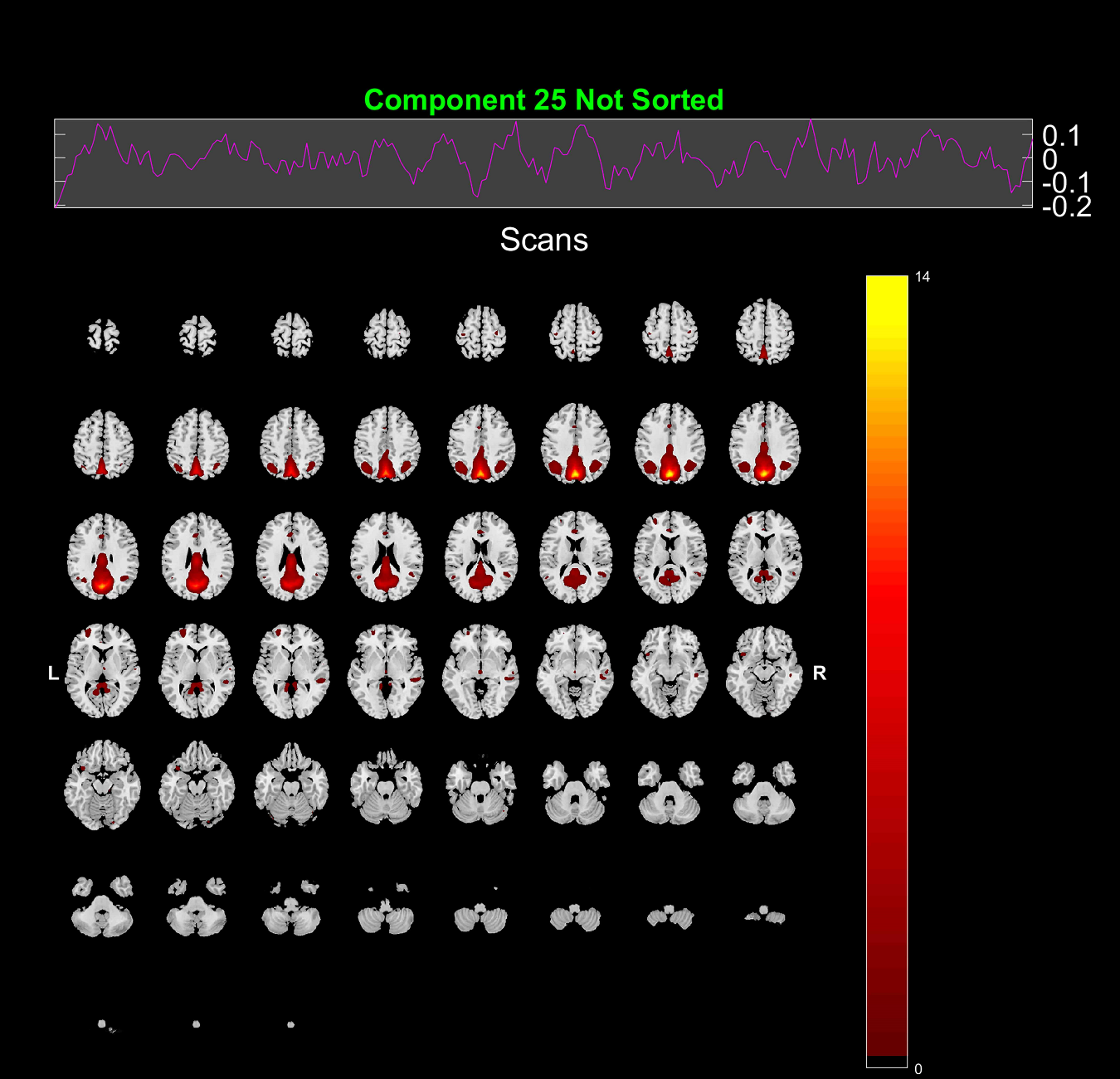


correlations with the DMN templates: r = 0.46176

correlations with the CEN templates: r = 0.0060465


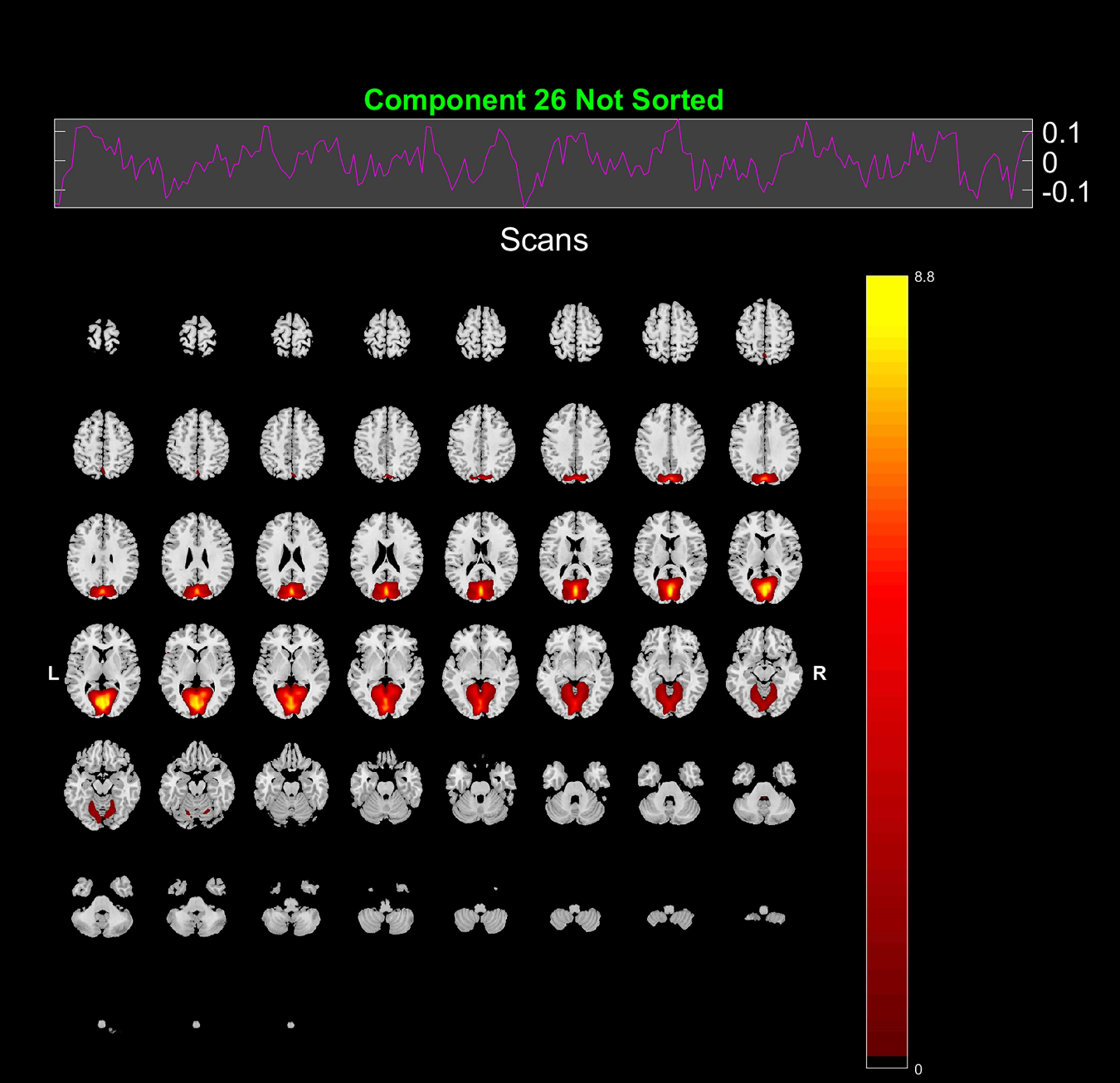


correlations with the DMN templates: r = 0.024047

correlations with the CEN templates: r = 0


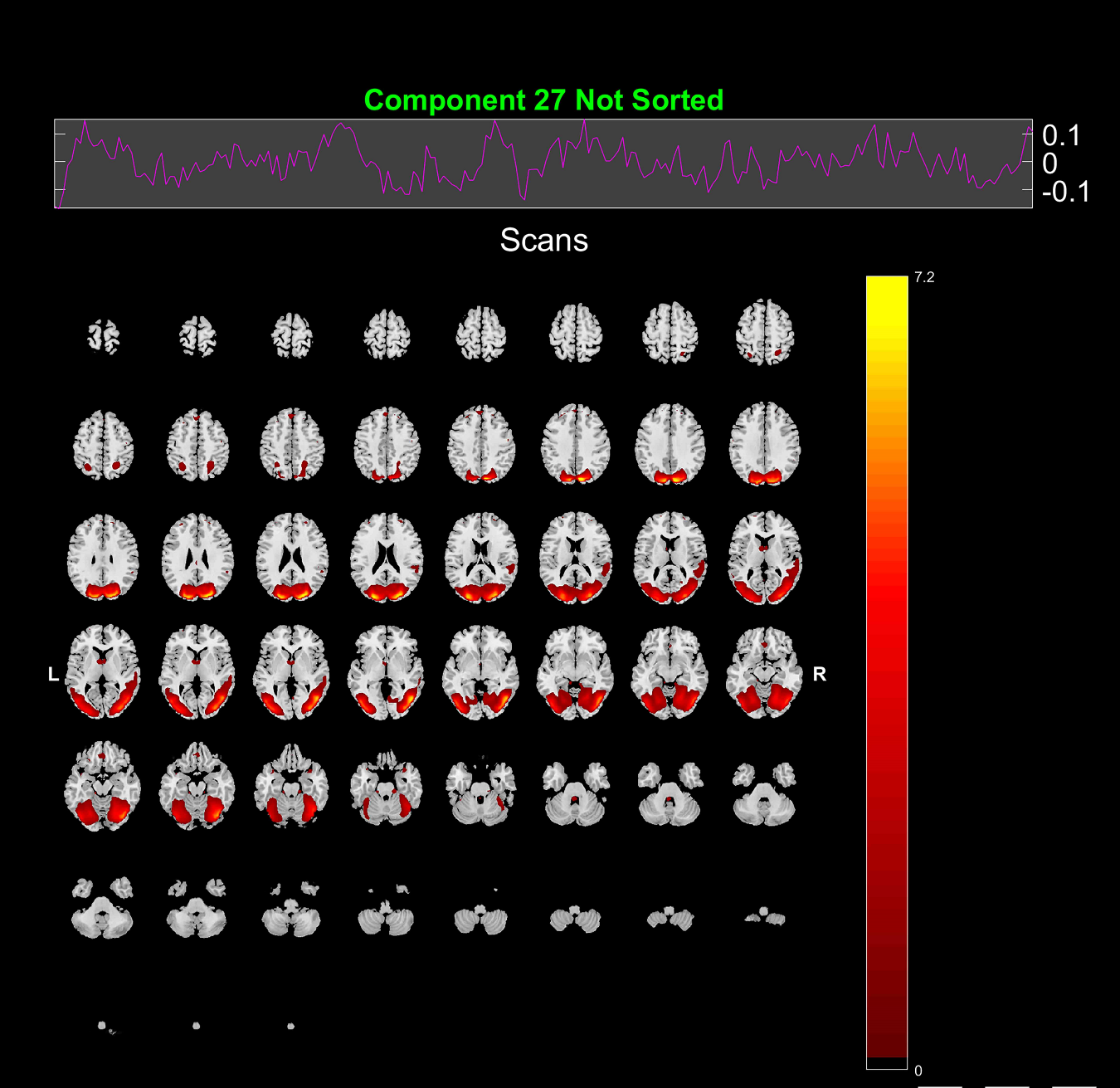


correlations with the DMN templates: r = -0.035255

correlations with the CEN templates: r = 0.032002

# References

**Cedres, N., Machado, A., Molina, Y., Diaz-Galvan, P., Hernandez-Cabrera, J. A., Barroso, J., Westman, E. & Ferreira, D.** (2019). Subjective Cognitive Decline Below and Above the Age of 60: A Multivariate Study on Neuroimaging, Cognitive, Clinical, and Demographic Measures. *J Alzheimers Dis* **68**, 295-309.

**Chen, J., Chen, G., Shu, H., Chen, G., Ward, B. D., Wang, Z., Liu, D., Antuono, P. G., Li, S. J., Zhang, Z. & Alzheimer's Disease Neuroimaging, I.** (2019a). Predicting progression from mild cognitive impairment to Alzheimer's disease on an individual subject basis by applying the CARE index across different independent cohorts. *Aging (Albany NY)* **11**, 2185-2201.

**Chen, J., Chen, R., Xue, C., Qi, W., Hu, G., Xu, W., Chen, S., Rao, J., Zhang, F. & Zhang, X.** (2022). Hippocampal-Subregion Mechanisms of Repetitive Transcranial Magnetic Stimulation Causally Associated with Amelioration of Episodic Memory in Amnestic Mild Cognitive Impairment. *J Alzheimers Dis* **85**, 1329-1342.

**Chen, J., Ma, N., Hu, G., Nousayhah, A., Xue, C., Qi, W., Xu, W., Chen, S., Rao, J., Liu, W., Zhang, F. & Zhang, X.** (2020). rTMS modulates precuneus-hippocampal subregion circuit in patients with subjective cognitive decline. *Aging (Albany NY)* **12**.

**Chen, J., Shu, H., Wang, Z., Zhan, Y., Liu, D., Liao, W., Xu, L., Liu, Y. & Zhang, Z.** (2016). Convergent and divergent intranetwork and internetwork connectivity patterns in patients with remitted late-life depression and amnestic mild cognitive impairment. *Cortex* **83**, 194-211.

**Chen, J., Shu, H., Wang, Z., Zhan, Y., Liu, D., Liu, Y. & Zhang, Z.** (2019b). Intrinsic connectivity identifies the sensory-motor network as a main cross-network between remitted late-life depression- and amnestic mild cognitive impairment-targeted networks. *Brain Imaging Behav*.

**Dunn, C. J., Duffy, S. L., Hickie, I. B., Lagopoulos, J., Lewis, S. J., Naismith, S. L. & Shine, J. M.** (2014). Deficits in episodic memory retrieval reveal impaired default mode network connectivity in amnestic mild cognitive impairment. *Neuroimage Clin* **4**, 473-80.

**Hao, L., Wang, X., Zhang, L., Xing, Y., Guo, Q., Hu, X., Mu, B., Chen, Y., Chen, G., Cao, J., Zhi, X., Liu, J., Li, X., Yang, L., Li, J., Du, W., Sun, Y., Wang, T., Liu, Z., Liu, Z., Zhao, X., Li, H., Yu, Y., Wang, X., Jia, J. & Han, Y.** (2017). Prevalence, Risk Factors, and Complaints Screening Tool Exploration of Subjective Cognitive Decline in a Large Cohort of the Chinese Population. *J Alzheimers Dis* **60**, 371-388.

**Jessen, F., Amariglio, R. E., van Boxtel, M., Breteler, M., Ceccaldi, M., Chetelat, G., Dubois, B., Dufouil, C., Ellis, K. A., van der Flier, W. M., Glodzik, L., van Harten, A. C., de Leon, M. J., McHugh, P., Mielke, M. M., Molinuevo, J. L., Mosconi, L., Osorio, R. S., Perrotin, A., Petersen, R. C., Rabin, L. A., Rami, L., Reisberg, B., Rentz, D. M., Sachdev, P. S., de la Sayette, V., Saykin, A. J., Scheltens, P., Shulman, M. B., Slavin, M. J., Sperling, R. A., Stewart, R., Uspenskaya, O., Vellas, B., Visser, P. J., Wagner, M. & Subjective Cognitive Decline Initiative Working, G.** (2014). A conceptual framework for research on subjective cognitive decline in preclinical Alzheimer's disease. *Alzheimers Dement* **10**, 844-52.

**Petersen, R. C., Smith, G. E., Waring, S. C., Ivnik, R. J., Tangalos, E. G. & Kokmen, E.** (1999). Mild cognitive impairment: clinical characterization and outcome. *Arch Neurol* **56**, 303-8.

**Winblad, B., Palmer, K., Kivipelto, M., Jelic, V., Fratiglioni, L., Wahlund, L. O., Nordberg, A., Backman, L., Albert, M., Almkvist, O., Arai, H., Basun, H., Blennow, K., de Leon, M., DeCarli, C., Erkinjuntti, T., Giacobini, E., Graff, C., Hardy, J., Jack, C., Jorm, A., Ritchie, K., van Duijn, C., Visser, P. & Petersen, R. C.** (2004). Mild cognitive impairment--beyond controversies, towards a consensus: report of the International Working Group on Mild Cognitive Impairment. *J Intern Med* **256**, 240-6.

**Xue, C., Yuan, B., Yue, Y., Xu, J., Wang, S., Wu, M., Ji, N., Zhou, X., Zhao, Y., Rao, J., Yang, W., Xiao, C. & Chen, J.** (2019). Distinct Disruptive Patterns of Default Mode Subnetwork Connectivity Across the Spectrum of Preclinical Alzheimer's Disease. *Front Aging Neurosci* **11**, 307.

**Yan, T., Wang, W., Yang, L., Chen, K., Chen, R. & Han, Y.** (2018). Rich club disturbances of the human connectome from subjective cognitive decline to Alzheimer's disease. *Theranostics* **8**, 3237-3255.
